# SARS-CoV-2 Vaccine-Induced T-Cell Response after Three Doses in People Living with HIV on Antiretroviral Therapy Compared to Seronegative Controls (CTN 328 COVAXHIV Study)

**DOI:** 10.3390/v15020575

**Published:** 2023-02-19

**Authors:** Yulia Alexandrova, Alexis Yero, Ralph-Sydney Mboumba Bouassa, Eve Comeau, Suzanne Samarani, Zabrina L. Brumme, Mark Hull, Angela M. Crawley, Marc-André Langlois, Jonathan B. Angel, Curtis L. Cooper, Judy Needham, Terry Lee, Joel Singer, Aslam H. Anis, Cecilia T. Costiniuk, Mohammad-Ali Jenabian

**Affiliations:** 1 Department of Biological Sciences and CERMO-FC Research Centre, Université du Québec à Montréal, Montreal, QC H2X 1Y4, Canada; 2 Infectious Diseases and Immunity in Global Health Program, Research Institute of McGill University Health Centre, Montreal, QC H4A 3J1, Canada; 3 Faculty of Health Sciences, Simon Fraser University, Burnaby, BC V5A 1S6, Canada; 4 British Columbia Centre for Excellence in HIV/AIDS, St. Paul’s Hospital, Vancouver, BC V6Z 1Y6, Canada; 5 Department of Medicine, University of British Columbia, Vancouver, BC V6T 1Z4, Canada; 6 Ottawa Hospital Research Institute, Ottawa, ON K1Y 4E9, Canada; 7 Department of Biochemistry, Microbiology and Immunology, University of Ottawa, Ottawa, ON K1H 8M5, Canada; 8 Coronavirus Variants Rapid Response Network–Biobank, Faculty of Medicine, University of Ottawa, Ottawa, ON K1H 8M5, Canada; 9 Centre for Infection, Immunity and Inflammation (CI3), University of Ottawa, Ottawa, ON K1H 8M5, Canada; 10 Department of Medicine, University of Ottawa, Ottawa, ON K1H 8M5, Canada; 11 CIHR Canadian HIV Trials Network and Centre for Health Evaluation and Outcome Sciences, St. Paul’s Hospital, Vancouver, BC V6Z 1Y6, Canada; 12 Department of Medicine, Division of Infectious Diseases and Chronic Viral Illness Service, McGill University Health Centre, Montreal, QC H4A 3J1, Canada

**Keywords:** COVID-19, SARS-CoV-2, HIV, PLWH, vaccine, T-cell immunity, cell-mediated immunity

## Abstract

People living with HIV (PLWH) may be at risk for poor immunogenicity to certain vaccines, including the ability to develop immunological memory. Here, we assessed T-cell immunogenicity following three SARS-CoV-2 vaccine doses in PLWH versus uninfected controls. Blood was collected from 38 PLWH on antiretroviral therapy and 24 age-matched HIV-negative controls, pre-vaccination and after 1st/2nd/3rd dose of SARS-CoV-2 vaccines, without prior SARS-CoV-2 infection. Flow cytometry was used to assess ex vivo T-cell immunophenotypes and intracellular Tumor necrosis factor (TNF)-α/interferon(IFN)-γ/interleukin(IL)-2 following SARS-CoV-2-Spike-peptide stimulation. Comparisons were made using Wilcoxon signed-rank test for paired variables and Mann–Whitney for unpaired. In PLWH, Spike-specific CD4 T-cell frequencies plateaued post-2nd dose, with no significant differences in polyfunctional SARS-CoV-2-specific T-cell proportions between PLWH and uninfected controls post-3rd dose. PLWH had higher frequencies of TNFα+CD4 T-cells and lower frequencies of IFNγ+CD8 T-cells than seronegative participants post-3rd dose. Regardless of HIV status, an increase in naive, regulatory, and PD1+ T-cell frequencies was observed post-3rd dose. In summary, two doses of SARS-CoV-2 vaccine induced a robust T-cell immune response in PLWH, which was maintained after the 3rd dose, with no significant differences in polyfunctional SARS-CoV-2-specific T-cell proportions between PLWH and uninfected controls post-3rd dose.

## 1. Introduction

Immune perturbations in both innate and adaptive immunity, chronic inflammation, accelerated immune aging, high prevalence of co-morbidities, and sociodemographic factors likely contribute to an increased risk of SARS-CoV-2 infection and complications in people living with HIV (PLWH) compared to persons without HIV [1,2]. Thus, achieving and maintaining optimal vaccine-induced T-cells is of particular importance for PLWH. Even despite suppressive antiretroviral therapy (ART), HIV infection may favor a poor serological response to vaccines, such as influenza and hepatitis B [3,4]. While some groups have reported that PLWH on ART show SARS-CoV-2-specific humoral and cellular responses that are similar to HIV-seronegative individuals [5,6], ongoing concerns remain regarding the magnitude and durability of immunological memory induced by SARS-CoV-2 vaccines in PLWH. Notably, despite the completion of a primary SARS-CoV-2 vaccination schedule (two doses of mRNA vaccine or one dose of adenoviral vector vaccine), PLWH were at higher risk of breakthrough infection than the general population [7]. The relative contribution of different factors contributing to increased SARS-CoV-2 infection risk, despite adequate vaccination, is still being investigated [2]. For instance, incomplete CD4 T-cell restoration and low CD4:CD8 ratios may impede the development of anti-SARS-CoV-2 immune responses following vaccination and infection, especially before booster vaccination [5,6,8]. Although we have recently shown that SARS-CoV-2 vaccine-induced humoral responses in PLWH on ART are not significantly different from HIV seronegative individuals [9], fewer studies have compared vaccine-induced SARS-CoV-2-specific T-cell immunity between PLWH and HIV seronegative individuals [6,10,11], of which only one has assessed responses after the 3rd dose [11]. Herein, we longitudinally assessed SARS-CoV-2-specific T-cell responses and changes in T-cell subset distributions in PLWH and HIV seronegative persons without prior SARS-CoV-2 infection, up to one month following the third SARS-CoV-2 vaccine dose.

## 2. Materials and Methods

### 2.1. Study Population

Cryopreserved peripheral blood mononuclear cells (PBMCs) from PLWH were obtained via the multi-centre prospective observational cohort study (CTN328) with recruitment sites located at the McGill University Health Center (Montreal, QC, Canada), University Health Network (Toronto, ON, Canada), the Ottawa Hospital (Ottawa, ON, Canada), and the British Columbia Centre for Excellence in HIV/AIDS (Vancouver, BC, Canada). Specimens from age-matched seronegative individuals were obtained from the Stop the Spread Ottawa (SSO) cohort [12]. Details regarding participant recruitment and inclusion/exclusion criteria for these cohorts have been previously described [8,12] and are outlined in Materials S1. For the current study, participants were excluded if they had received any blood product or immunoglobulin preparation within 1 month of vaccination or if they experienced a SARS-CoV-2 infection (according to nucleic acid-, antigen-, or serology-based tests) during follow-up. Participants with specimens available at the time points of interest were identified from each cohort, and age and sex matched as closely as possible.

### 2.2. Vaccine Doses and Study Visits

**Figure 1:**
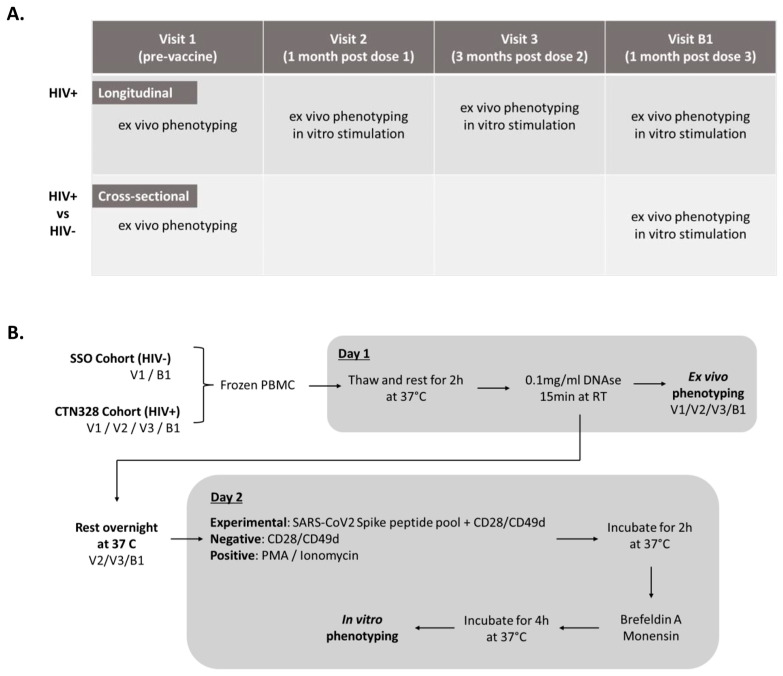
Experimental setup. (**A**) Longitudinal analysis was performed on blood cells collected during Visit 1 (V1; pre-vaccination), Visit 2 (V2; 1 month post 1st dose), Visit 3 (V3; 3 months post 2nd dose), and Visit B1 (B1; 1 month post 3rd dose) in people living with HIV (PLWH). Cross-sectional comparisons between PLWH and seronegative controls were made on samples collected at Visits 1 and B1 only. (**B**) Frozen PBMCs were thawed, rested at 37 °C for 2 h, incubated with DNAse I for 15 min at RT, and stained with ex vivo flow cytometry phenotyping panels. In total, 10^6^ PBMCs per experimental condition were rested at 37 °C overnight and then stimulated with CD28/CD49d co-stimulatory antibodies in presence or absence of SARS-CoV2 Spike peptides for 6 h. Each sample batch included two positive controls stimulated with PMA/Ionomycin. Brefeldin A and Monensin were added 4 h before the end of incubation.

We analyzed samples from 38 PLWH at baseline (prior to vaccination; Visit 1), 1 month post 1st dose (Visit 2), 3 months post 2nd dose (Visit 3), and 1 month post 3rd dose (visit B1). For the 24 age-matched controls, only pre-vaccination (Visit 1) and a three-month post-3rd dose (Visit B1) samples were available, so comparisons between PLWH and controls were restricted to these time points (Figure 1A).

### 2.3. Ex Vivo Immunophenotyping of T-Cells

Ex vivo extracellular staining was performed in PBS + 2% fetal bovine serum for 1 h at 4 °C. The cells were then fixed and permeabilized using the transcription factor buffer Set according to manufacturer’s protocol (BD Bioscience, Mississauga, ON, Canada), incubated with the appropriate antibodies in a Perm/Wash buffer for 1 h at 4 °C, and acquired on a BD Fortessa-X20. Antibodies used for phenotyping are listed in Table S1. All flow cytometry data was analyzed using FlowJo V10.8.1.

### 2.4. Intracellular Cytokine Production upon In Vitro SARS-CoV-2 Peptide Stimulation

**Figure 2:**
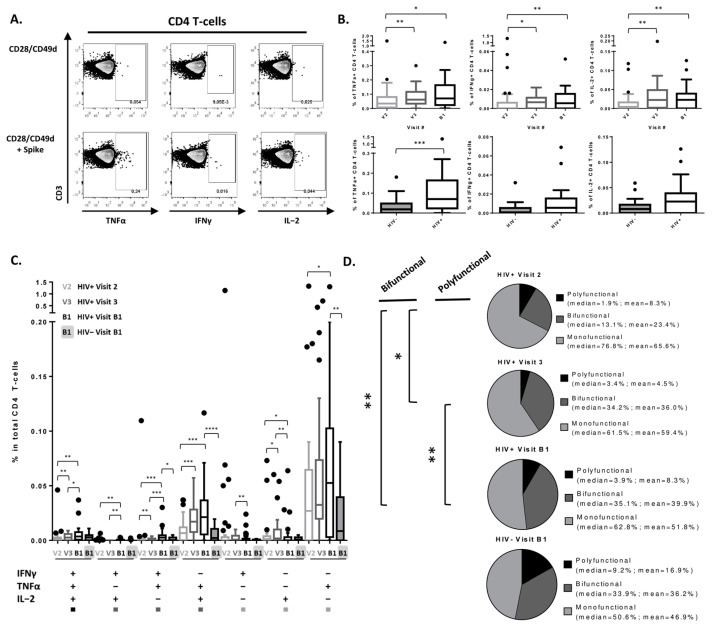
SARS-CoV-2-specific CD4 T-cell response. (**A**) Examples of flow cytometry plots of live CD4 T-cells with gates on TNFα+, IFNγ+, and IL-2+ cells from left to right, respectively. Signal detected in “CD28/CD49d” was subtracted from “CD28/CD49d + Spike” for all samples. (**B**) Frequencies of TNFα+, IFNγ+, and IL-2+ CD4 T-cells post 1st, 2nd, and 3d vaccine doses in PLWH (top row) and post 3d vaccine dose in PLWH versus seronegative controls with (bottom row). Lines in HIV+ versus HIV- plots indicated the median and interquartile range. (**C**) Frequencies of cytokine producing SARS-CoV-2-specific CD4 T-cells in total CD4 T-cell pool at different time points in PLWH and uninfected controls. Squares at the bottom of the graph indicate whether the cell subset was classified as polyfunctional, bifunctional, or monofunctional when graphing the pie chart on Figure 2D. (**D**) Proportions of polyfunctional, bifunctional, and monofunctional cells within SARS-CoV-2-specific CD4 T-cells at different time points in PLWH and uninfected controls. Median frequencies of each subset in SARS-CoV-2-specific CD4 T-cells are indicated to the right of each pie chart. Pie charts were generated using the mean values. Comparisons were made using Wilcoxon-Pratt signed rank test for paired variables and Mann–Whitney for unpaired variables (* *p* < 0.05, ** *p* < 0.01, *** *p* < 0.001, **** *p* < 0.0001). Study visits: V1—pre-vaccination baseline, V2—1 month post 1st dose, V3—3 months post 2nd dose, B1—1 month post 3rd dose.

**Figure 3:**
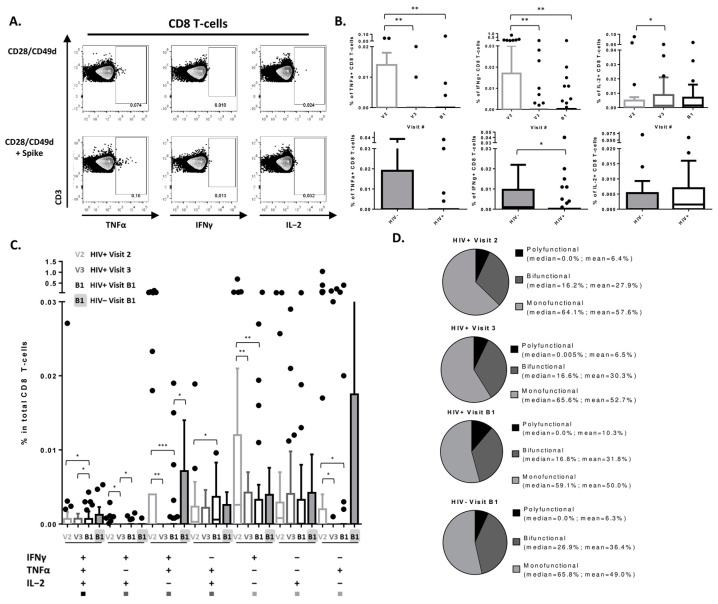
SARS-CoV-2-specific CD8 T-cell response. (**A**) Examples of flow cytometry plots of live CD8 T-cells with gates on TNFα+, IFNγ+, and IL-2+ cells from left to right, respectively. Signal detected in “CD28/CD49d” was subtracted from “CD28/CD49d + Spike” for all samples. (**B**) Frequencies of TNFα+, IFNγ+, and IL-2+ CD8 T-cells post 1st, 2nd, and 3d vaccine doses in PLWH (top row) and post 3d vaccine dose in PLWH versus seronegative controls with (bottom row). Lines in HIV+ versus HIV- plots indicated the median and interquartile range. (**C**) Frequencies of cytokine producing SARS-CoV-2-specific CD8 T-cells in total CD8 T-cell pool at different time points in PLWH and uninfected controls. Squares at the bottom of the graph indicate whether the cell subset was classified as polyfunctional, bifunctional, or monofunctional when graphing the pie chart on Figure 2D. (**D**) Proportions of polyfunctional, bifunctional, and monofunctional cells within SARS-CoV-2-specific CD8 T-cells at different time points in PLWH and uninfected controls. Median frequencies of each subset in SARS-CoV-2-specific CD8 T-cells are indicated to the right of each pie chart. Pie charts were generated using the mean values. Comparisons were made using Wilcoxon-Pratt signed rank test for paired variables and Mann–Whitney for unpaired variables (* *p* < 0.05, ** *p* < 0.01, *** *p* < 0.001). Study visits: V1—pre-vaccination baseline, V2—1 month post 1st dose, V3—3 months post 2nd dose, B1—1 month post 3rd dose.

In vitro stimulation was performed with CD28/CD49d co-stimulatory antibodies (2 ug/mL) (BD Bioscience, Mississauga, ON, Canada) in the presence or absence of SARS-CoV-2 Spike peptides (0.6 ug of each peptide/mL) (PepTivator SARS-CoV-2 Prot_S, Miltenyi Biotec, Bergisch Gladbach, Germany) in RPMI 10% Human Serum Type AB (Wisent, Saint-Jean-Baptiste, QC, Canada) at 37 °C (Figure 1B). Monensin (2 uM) (BD Bioscience, Mississauga, ON, Canada) and Brefeldin-A (1 ug/mL) (BD Bioscience, Mississauga, ON, Canada) were added 2 h after stimulation initiation and incubated for an additional 4 h. After stimulation, cells were washed and stained extracellularly with anti-human CD3/CD4/CD8 antibodies and Live/Dead. Cells were then washed and stained intracellularly with anti-human TNFα/IFNγ/IL-2 antibodies for 30 min at room temperature using the Cytofix/Cytoperm kit (BD Bioscience, Mississauga, ON, Canada). Antibodies used for in vitro analysis are listed in Table S2. The SARS-CoV-2 specific responses were calculated as the delta between the Spike-stimulated condition versus CD28/CD49d-only stimulation (Figure 2A and Figure 3A).

### 2.5. Ethical Statement

All participants provided written informed consent for this study (Materials S1). Ethical approval for this CTN 328 sub-study was obtained from UQAM ethical board (#2022-4615).

### 2.6. Statistical Analyses

Clinical data were analyzed using R version 4.2.2 statistical software (R Foundation for Statistical Computing, Vienna, Austria). Continuous variables are presented as the median and interquartile range (IQR), whereas categorical variables are shown as numbers and percentages. Statistical analyses of immunogenicity data were performed using GraphPad Prism V6.01 (San Diego, CA, USA). Cross-sectional comparisons between groups were made using χ2, Fisher’s exact test or the Mann–Whitney U test. Pairwise comparisons between time points were performed using the Wilcoxon signed-rank test for ex vivo analyses and Wilcoxon-Pratt for in vitro analyses. Strength and directions of associations between two variables were measured using Spearman’s rank correlation. *p*-values < 0.05 were considered statistically significant.

## 3. Results

### 3.1. Study Population

For this CTN328 sub-study, 38 PLWH and 24 seronegative controls were included, with demographics outlined in Table 1. Groups were similar in terms of age, although a greater proportion of PLWH were males and fewer were Caucasian, compared with the seronegative controls. All participants were negative for Hepatitis B and C infection.

### 3.2. Peak SARS-CoV-2-Specific CD4 T-Cell Cytokine Secretion Response Achieved after 2nd Vaccine Dose in PLWH

In PLWH, frequencies of SARS-CoV-2-specific TNFα+, IFNγ+, and IL-2+ CD4 T-cells peaked after the second vaccine dose and plateaued thereafter, with no significant differences between Visits 3 and B1 (V2 vs. V3: TNFα *p* = 0.008/IFNγ *p* = 0.03/IL-2: *p* = 0.002; V2 vs. B1: TNFα *p* = 0.01/IFNγ *p* = 0.0095/IL-2 *p* = 0.004; Figure 2B). PLWH also showed higher frequencies of TNFα+ CD4 T-cells post-3rd dose compared to seronegative controls (*p* = 0.0009). Notably, when the same analyses were performed on participants who received mRNA vaccine doses only, frequencies of IL-2+ CD4 T-cells also became significantly higher in PLWH versus seronegative individuals (*p* = 0.03) (Supplementary Figure S1). We then further stratified SARS-CoV-2-specific T-cells based on their cytokine secretion profile, where cells secreting all three measured cytokines were classified as polyfunctional, two of the three cytokines as bifunctional, and only one of the three cytokines as monofunctional. Frequencies of polyfunctional and bifunctional cells within total CD4 T-cells increased significantly after the 2nd vaccine dose and were maintained after the 3rd dose (Figure 2C). Notably, these subsets were largely TNFα+ cells co-expressing other cytokines. Similarly, within SARS-CoV-2-specific CD4 T-cells, the proportion of bifunctional cells increased significantly after the 2nd vaccine dose (V2 vs. V3: *p* = 0.02; V2 vs. B1: *p* = 0.005; V3 vs. B1: *p* = 0.66) and of polyfunctional after the 3rd dose (V2 vs. V3: *p* = 0.2; V2 vs. B1: *p* = 0.2; V3 vs. B1: *p* = 0.009) in PLWH (Figure 2D). There were no significant differences in proportions of polyfunctional, bifunctional, or monofunctional cells between PLWH and seronegative controls after the 3rd vaccine dose (Figure 2D). Similar results were observed when participants who received the Astrazeneca vaccine as their 1st dose were excluded from analyses (Supplementary Figure S2). Lastly, we found no significant correlation between CD4 T-cell response and participants’ clinical features (Supplementary Table S3). To assess the potential impact of ART regimen on CD4 polyfunctionality, HIV-positive participants on INSTI-based regimen versus other ART regimens were compared and no significant differences have been observed (data not shown).

### 3.3. SARS-CoV-2 Vaccine Induces a Detectable CD8 T-Cell Response in PLWH

SARS-CoV-2 vaccination induced an antigen-specific CD8 T-cell response that was detectable in our in vitro stimulation assay, albeit it was less prominent than in CD4 T-cells. A decrease in frequencies of TNFα+ and IFNγ+ SARS-CoV-2-specific CD8 T-cells (V2 vs. V3: TNFα *p* = 0.008, IFNγ *p* = 0.004; V2 vs. B1: TNFα *p* = 0.003, IFNγ *p* = 0.002) were observed after the 1st vaccine dose in PLWH (Figure 3B). Interestingly, frequencies of IL-2+ CD8 T-cells increased after the 2nd vaccine dose (V2 vs. V3: *p* = 0.03) and remained unchanged after the 3rd dose. Furthermore, levels of IFNγ+ CD8 T-cells were significantly lower in PLWH compared to controls (*p* = 0.02) and showed a significant positive correlation with the CD4:CD8 ratio (*r =* 0.372; *p* = 0.047) (Supplementary Table S4). There were no significant differences in the SARS-CoV-2-specific CD8 T-cell polyfunctionality profile between PLWH and controls (Figure 3C,D). Similar results were observed when participants who received the Astrazeneca vaccine as their 1st dose were excluded from analyses (Supplementary Figures S1 and S2). To assess the potential impact of the ART regimen on CD8 polyfunctionality, HIV-positive participants on the INSTI-based regimen versus other ART regimens were compared and no significant differences have been observed (data not shown).

### 3.4. Changes in CD4 T-Cell Subsets following 3 Doses of SARS-CoV-2 Vaccination

Frequencies of naïve CD4 T-cells (CD45RA+CD28+CCR7+) increased post-vaccination, regardless of HIV status, with significantly higher naive cell frequencies observed in PLWH versus controls. Frequencies of transitional memory (CD45RA-CD28+CCR7-), effector memory (CD45RA-CD28-CCR7-), and terminally differentiated (CD45RA+CD28-CCR7-) cells decreased over time in both groups. Lower levels of central memory CD4 T-cells were observed in PLWH versus controls but without any significant change post-vaccination compared to baseline (Table 2). In line with increased naïve CD4 T-cell frequencies, levels of senescent (CD57+CD28-) CD4 T-cells and Ki67+ CD4 T-cells decreased in both groups over time. Activated CD4 T-cell levels (HLA-DR+CD38+) decreased over time in PLWH. Furthermore, frequencies of PD-1+ CD4 T-cells increased post-vaccination, regardless of HIV status, with higher expression levels observed in PLWH compared to controls. Levels of CTLA-4+ CD4 T-cells decreased significantly in controls from Visit 1 to B1, but not in PLWH. Levels of CD39+ CD4 T-cells increased in controls from V1 to B1 but not in PLWH, with significantly higher frequencies in the HIV+ study group. An opposite trend was observed for CD73 expression, which increased in CD4 T-cells from PLWH but not in controls.

Within total CD4 T-cells, we observed a significant increase in CCR4+ cell frequencies and a significant decrease in CXCR3+ cell frequencies, with no change in CCR6 expression regardless of HIV status over time post-vaccination (Table 2). A similar pattern in Th subsets was observed. Frequencies of Th1 cells decreased, while frequencies of Th2 and Th17 cells increased over time in both groups from Visit 1 to B1. Additionally, frequencies of total Tregs, CD39+ Tregs, and CD73+ Tregs also increased over time in both groups post-vaccination.

Similar results were observed when participants who received the Astrazeneca vaccine as their 1st dose were excluded from analyses (Supplementary Table S5). Gating examples on CD4 T-cell phenotypical markers are shown in Supplementary Figures S3 and S4.

### 3.5. Changes in CD8 T-Cell Subsets following 3 Doses of SARS-CoV-2 Vaccination

Similarly to CD4 T-cells, we observed increased levels of naïve and reduced levels of effector memory and terminally differentiated CD8 T-cell subsets post-vaccination, regardless of HIV status (Table 3). However, in contrast to CD4 T-cells, we saw significantly higher levels of central memory and transitional memory CD8 T-cells in both PLWH and controls. In line with this observation, frequencies of senescent cells decreased over time in both groups. Increased PD-1 and decreased Ki67 expression was observed over time in both groups. There were no significant differences in CTLA-4 expression post-vaccination. Frequencies of CD73+ CD8 T-cells increased over time after vaccination, regardless of HIV status, with significantly lower levels observed in PLWH versus controls. CD39 expression in CD8 T-cells was significantly lower in PLWH than in controls and showed no significant changes over time. However, a significant increase in CD39+ CD8 T-cells was observed in controls post-3rd dose. Lastly, frequencies of CCR4+ and CXCR3+ CD8 T-cells decreased post-vaccination, regardless of HIV status. No significant changes in frequencies of CCR6+ CD8 T-cells were observed.

Similar results were observed when participants who received the Astrazeneca vaccine as their 1st dose were excluded from analyses (Supplementary Table S6). Gating examples on CD4 T-cell phenotypical markers are shown in Supplementary Figure S5.

## 4. Discussion

Herein, we have demonstrated that three doses of SARS-CoV-2 vaccine induced a detectable Spike-specific T-cell IFNγ/TNFα/IL2 cytokine response in PLWH. Peak SARS-CoV-2-specific CD4 T-cell responses were achieved after the 2nd vaccine dose and were maintained after the 3rd dose. Previous studies have shown that PLWH on ART mount detectable cellular and humoral responses after SARS-CoV-2 infection [5]. This is also the case for SARS-CoV-2 vaccination [6,10,13], although most of these studies have only assessed IFNγ production after one or two SARS-CoV-2 vaccine doses. However, in our study, we analyzed intracellular levels and production patterns of three different cytokines in CD4 and CD8 T-cells, excluding innate immune cells. This strategy differs from the techniques employed in the aforementioned studies, which measure cytokine concentration directly in the supernatant, since they do not discriminate between innate and adaptive cytokine origins. To our knowledge, there has only been one recent study that looked at SARS-CoV-2-specific T-cells in 8 PLWH after the fourth vaccine dose, demonstrating a detectable humoral and cellular response [14]. Our current study adds new knowledge to this collective body of work, looking at longitudinal dynamics of SARS-CoV-2-specific T-cells prior to 1st, 2nd, and 3rd doses and how they compare to HIV-uninfected individuals.

CD4 T-cell counts and CD4:CD8 ratio are major predictors of immune response efficacy, where CD4 counts under 200/mm^3^ are linked with a lower magnitude of anti-spike and neutralizing antibodies, a poor IFN-γ response, and an increased risk of severe illness despite vaccination [6,15]. In our PLWH group, all participants had CD4 T-cell counts >200/mm^3^ (median of 700/mm^3^) with comparable anti-SARS-CoV-2 T-cell responses to controls. Furthermore, we show a significant positive correlation between levels of IL-2+ CD8 T-cells and the CD4:CD8 ratio. This is in line with previously published data in the context of natural- and vaccine-induced anti-SARS-CoV-2 T-cell immunity [5,6]. Indeed, PLWH who respond well to ART and display adequate immune reconstitution, usually defined as a CD4 count ≥350 cells/mm^3^, also show SARS-CoV-2-specific T-cell responses that are similar to individuals without HIV [5,6]. We also demonstrated that there were no significant differences in the proportions of polyfunctional T-cell subsets between PLWH and HIV-uninfected individuals post-3rd dose. To our knowledge, similar analyses have only been reported in one study in the context of SARS-CoV-2 natural infection [5], but not after three SARS-CoV-2 vaccinations in SARS-CoV-2 infection-naïve PLWH.

We found significantly higher frequencies of TNFα-producing Spike-specific CD4 T-cells in PLWH compared to controls. Elevated T-cell production of pro-inflammatory cytokines, such as TNFα, in chronic HIV infection despite suppressive ART has been well characterized [16,17,18,19]. Whether higher frequencies of TNFα+ CD4 T-cells are associated with better or worse vaccine-induced protection against severe illness in PLWH requires further investigation. We also report significantly lower frequencies of IFNγ-producing Spike-specific CD8 T-cells in PLWH versus controls, along with an overall decrease in both IFNγ and TNFα production over time in PLWH. This finding is expected since chronic HIV infection is known to cause persistent immune stimulation and subsequent CD8 T-cell exhaustion [20]. Exhausted CD8 T-cells, in turn, exhibit impaired cytokine production, such as IFNγ and TNFα, and poor anti-viral function [20,21]. Similarly, in our ex vivo data we observed increased frequencies of PD-1+ CD8 T-cells at baseline in PLWH versus seronegative controls, which could explain their reduced IFNγ secretion upon SARS-CoV-2-Spike peptide stimulation. However, the differences in medians in SARS-CoV-2-specific CD8 T-cells between PLWH and controls is extremely small (0.001%) and whether this has a direct impact on SARS-CoV-2 vaccine efficacy in PLWH on ART remains to be elucidated. Of note, our methods of in vitro peptide stimulation, which has been widely used by other study groups [5,22], could favor MHC class II presentation over MHC Class I, which could result in the increased detection of SARS-CoV-2-specific CD4 rather than CD8 T-cells.

Notably, despite the initially non-significant differences that were observed in CD4 T-cell IL-2 production between HIV+ and HIV- groups (Figure 2), when participants who received an adenovirus-based vaccine (Astrazeneca) as their 1st dose were excluded from analyses, frequencies of IL-2+ CD4 T-cells became significantly higher in PLWH versus seronegative individuals (Supplementary Figure S1). This comes as no surprise, since previous reports have shown that heterologous dosing with an adenovirus-based vaccine followed by an mRNA vaccine results in more-robust cellular immunity than homologous vaccination [23]. Because all heterologously vaccinated participants in this study came from the HIV- group, it is expected that data points with higher frequencies of cytokine-positive cells would be lost. Furthermore, 38% of participants in our HIV+ study group (*n* = 14) were mRNA-1273 (Moderna) vaccinees across all time points, and only 4% in the HIV- group (*n* = 1). Zhang et al. have recently demonstrated that mRNA-1273 vaccinees had significantly higher frequencies of TNFα+ and IL-2+ CD4+ T-cells relative to those who received the BNT162b2 vaccine (Pfizer), which could help explain our observations [24].

Regardless of HIV status, we observed a progressive increase in naïve CD4 and CD8 T-cell frequencies after SARS-CoV-2 vaccination. Previous groups have reported a positive correlation between SARS-CoV-2-specific T-cell response and naïve CD4 T-cell frequencies in PLWH [5]. Similarly, we observed a significant positive correlation between IL-2+ SARS-CoV-2-specific CD4 T-cells and naïve CD4 T-cell frequencies in PLWH (Spearman *r* = 0.32; *p* = 0.047). Higher frequencies of naïve T-cells have been linked with better COVID-19 outcomes not only in PLWH, but also in the general population, including children, and naïve T-cells are a key component of COVID-19 severity [25,26]. Importantly, factors which have been linked with worse COVID-19 outcomes, such as old age, male sex, and diabetes, have also been linked with lower naïve T-cell production [26,27]. A higher percentage of naïve T-cells and higher thymic function has also been associated with a higher SARS-CoV-2 vaccine response, both of which are decreased in vaccinees over the age of 60 [27]. Thus, higher naïve T-cell numbers and greater TCR diversity maximize the chance of the immune system to recognize a new pathogen and mount an effective adaptive immune response [5,25,26]. Therefore, increased frequencies of naïve T-cells after each subsequent SARS-CoV-2 vaccination are likely a positive sign, indicating increased thymic output and a higher likelihood of the generation of a successful vaccine-induced T-cell response to SARS-CoV-2. In line with this observation, it is well known that naïve T-cells express less Ki67 and do not express markers of cell senescence compared to memory T-cell subsets in both PLWH and uninfected individuals [28,29]. Thus, it was unsurprising that frequencies of Ki67+ and CD57+CD28- senescent cells in total CD4 and CD8 T-cells decreased over time in both study groups. Similarly, we observed a significant decrease in HLA-DR+CD38+ CD4 T-cells in PLWH post-vaccination. However, this decrease was not significant in seronegative controls. This is likely due to significantly higher naïve CD4 T-cell frequencies in PLWH at baseline and after vaccine administration. Because HLA-DR and CD38 are also largely expressed by memory rather than naïve T-cells, it would be expected for HLA-DR+CD38+ cell frequencies to decrease in the total CD4 T-cell pool if the proportion of the naïve subset increases [30].

In PLWH, we observed a significant increase in CCR4+ CD4 T-cells over time post-vaccination. CCR4 is a chemokine receptor expressed by Th2 and Treg cell subsets [31] that is also implicated in lung homing [32]. Notably, we observed an increase in both Th2 and Treg cell subsets, and a decrease in the Th1 subset, and its corresponding chemokine receptor CXCR3, regardless of HIV status. This observation may seem contradictory to those reported in previous studies, which demonstrated that vaccine-induced SARS-CoV-2 specific T-cells belong primarily to the Th1 subset [33,34]. Importantly, however, in our ex vivo phenotyping studies, we evaluated total CD4 and CD8 T-cell pools, assessing global effects of the vaccine on the entire T-cell population. This differs from our in vitro stimulation where we see an increase in Th1 cells based on Spike-induced cytokine secretion, since in our ex vivo phenotyping data we evaluated total CD4 and CD8 T-cell pools, assessing global effects of the vaccine on the entire T-cell population. Additionally, the overall increase in Th2 and Th17 and decrease in Th1 cells after vaccination might be mediated by increased Treg frequencies and function. Levine and colleagues have previously demonstrated that murine Tbet+ Tregs specifically suppress Th1 and CD8 T-cell responses while not affecting Th2 or Th17 cells [35]. Furthermore, they show that these Tregs expand during a recall response to *Listeria monocytogenes*, which is an intracellular pathogen that induces a Type 1 immune response under normal conditions [35]. Increased Treg frequencies and naïve CD8 T-cell subsets could also explain why we observed reduced frequencies of activated HLADR+CD38+ CD8 T-cells [36,37]. Interestingly, lower frequencies of CXCR3+ and CCR4+ CD8 T-cells post-3rd dose versus baseline could be in part explained by an increase in central and transitional memory CD8 T-cell subsets that we have observed. Some studies have proposed that CXCR3 is involved in promoting CD8 T cell commitment to an effector rather than memory subsets via their recruitment to inflammatory sites [38]. The absence of CXCR3 expression on CD8 T cells can lead to the formation of more memory precursors and fewer short-lived effectors [38]. Some groups also reported that CCR4 supports the generation of short-lived effector CD8 T-cells and that there is a significant correlation between CCR4 expression on CD8 T-cells and their increased effector differentiation in HIV-negative adults with severe COVID-19 [32,39].

Increased Treg frequencies could have both beneficial and detrimental effects on SARS-CoV-2 vaccine-induced T-cell immunity. On one hand, Tregs are known for inhibiting vaccine-induced T-cell response [40]. On the other hand, they can prevent severe COVID-19 immunopathology [41]. In the context of SARS-CoV-2 vaccination, increased Treg levels accompanied by effective T-cell and B-cell responses could prevent severe inflammatory pathology [41]. However, they could also be part of the reason underlying the need for frequent SARS-CoV-2 vaccine boosters to maintain protection against severe illness. Interestingly, Treg depletion in mice has been shown to be associated with long lasting vaccine-induced memory [42,43].

CD39 and CD73 ectonucleotidases are T-cell activation markers that act as immunological switches by converting pro-inflammatory extracellular adenosine triphosphate into immunosuppressive adenosine [44]. In addition to Tregs, expression levels of CD73 were increased in total CD4 T-cells after vaccination, regardless of HIV status, with significantly lower levels in PLWH. Furthermore, levels of CD39 increased in seronegative controls after the 3rd dose but not in PLWH, in whom CD39 expression was already significantly elevated across all time points, likely due to HIV infection [45]. In CD8 T-cells, expression levels of CD73 increased over time, regardless of HIV status, with higher frequencies observed in seronegative controls. This is also a positive sign, since CD73 expression on memory CD8 T-cells is correlated with reduced cell activation, spontaneous control of HIV replication in HIV controllers, and improved HIV-specific CD8 T-cell expansion [46]. In our data, we observed increased levels of CD39 in controls, but not in PLWH, who also displayed significantly lower levels of this molecules at baseline and post-3rd dose. Although high levels of CD39 on CD8 T-cells is indicative of terminal exhaustion in chronic viral infections, it might not be applicable to CD8 T-cells from donors without such infections [47]. Furthermore, CD39 expression can mark CD8 T-cells that produce high levels of IFNγ [48], which might explain why we see significantly higher levels of IFNγ+ SARS-CoV-2-specific CD8 T-cells in controls compared to PLWH.

Levels of CTLA-4 were higher in PLWH at baseline than in controls, although this difference was not statistically significantly (*p* = 0.058) and became nearly indistinguishable after the 3rd vaccine dose (*p* = 0.9). CTLA-4 expression is upregulated in response to inflammation and its levels are already elevated during HIV infection [49], which is likely why we see its significant upregulation in controls after the 3rd dose but not in PLWH. Lastly, we observed increased PD-1 expression on both CD4 and CD8 T-cells regardless of HIV-status. Similar to CTLA-4, PD-1 is transiently upregulated in response to T-cell receptor (TCR) triggering. While it is typically known as a T-cell exhaustion marker linked to poor T-cell function in PLWH, its primary function is the regulation of T-cell activation to prevent tissue damage [50]. Interestingly, some studies have suggested that higher PD-1 expression on activated CD8 T-cells might help prevent immune hyperactivation [51,52]. This is supported by a previous study showing that hyperactivated SARS-CoV-2 specific CD8 T-cells display impaired exhaustion levels during severe COVID-19 [51]. Whether PD-1+ T-cells should be categorized as exhausted in the context of SARS-CoV-2 is controversial. Some studies have argued that PD-1+ SARS-CoV-2-specific CD8 T-cells during SARS-CoV-2 infection remain highly functional, expressing higher levels of IFNγ than their PD-1- counterparts [53]. Thus, in our study, it would be more accurate to interpret PD-1 expression as a marker of TCR activation given the increase in SARS-CoV-2-specific T-cell cytokine secretion that we have observed. This finding may appear to contrast with the decrease in frequencies we have observed in other activation markers previously mentioned, including HLA-DR and CD38, which can be attributed to the increase in naïve T-cells. However, unlike HLA-DR/CD38, previous studies have shown that a naive T-cell can also express PD-1 and that its blockade interferes with their transition to effector memory phenotype, which would explain our observations [54,55,56].

There are some limitations to our study. Inherent differences in CTN328 and SSO cohorts posed difficulties during participant matching. We could not obtain matching time points after the 1st and 2nd vaccine doses for controls and there were some discrepancies in biological sex and ethnicity between groups. Being male is considered a risk factor for severe COVID-19 [57], and some studies report that there is an association between increased levels of SARS-CoV-2-specific T-cells and male sex [58]. Others report that there is no correlation between SARS-CoV-2-specific cellular immunity and biological sex in both PLWH and uninfected persons [5]. Of note, none of the participant characteristics (age, sex, ethnicity) showed any significant correlation with SARS-CoV-2-specific T-cell frequencies in our analyses, so it is unlikely that imperfect sex and ethnicity matching has affected our cross-sectional comparisons. Finally, a relatively small sample size might also result in insufficient statistical power in some comparisons.

In summary, we report that two doses of COVID-19 vaccine induced a robust T-cell immune response to the SARS-CoV-2 Spike protein in PLWH, which was maintained after the 3rd dose. Further research is needed to determine the duration of this response to inform the recommended timing of additional vaccine doses in PLWH.

## Figures and Tables

**Table 1:** Participant characteristics. INSTI = integrase strand inhibitor; NNRTI = non-nucleoside r verse transcriptase inhibitor; NRT = nucleoside reverse transcriptase inhibitor. Significant differences (*p* < 0.05) are highlighted in bold.

Characteristics	PLWH, N = 38 ^1^	Uninfected, N = 24 ^1^	*p*-Value
Age, years, median (IQR)	43 (36, 57)	44 (38, 56)	0.8 ^2^
Male sex, n (%)	33 (87%)	12 (50%)	**0.002** ^3^
Ethnicity, n (%)			**0.008** ^4^
Caucasian	21 (55%)	24 (100%)	
Black	2 (5.3%)	0 (0%)	
Chinese	4 (11%)	0 (0%)	
Filipino	2 (5.3%)	0 (0%)	
Japanese	1 (2.6%)	0 (0%)	
Latin American	5 (13%)	0 (0%)	
Other	3 (7.9%)	0 (0%)	
SARS-CoV2 vaccine type received, n (%)			**0.019** ^4^
3 doses of mRNA (Pfizer or Moderna)	38 (100%)	20 (83%)	
1st dose Adenovirus vector (Astrazeneca), 2nd/3d dose mRNA (Pfizer or Moderna)	0 (0%)	4 (17%)	
CD4 count (n = 35), cells/mm^3^, median (IQR)	700 (480, 839)	N/A	N/A
CD4/CD8 ratio (n = 35)	0.81 (0.59, 1.01)		
CD4 nadir, cells/mm^3^, median (IQR) (n = 37)	290 (167, 420)		
Duration of HIV infection, years (IRQ) (n = 37)	6.5 (5.0, 21.0)		
Undetectable viral load for at least 6 months, n (%)	30 (79%)		
If detectable, highest viral load over past 6 months (n = 8), median (IQR) (copies/mL)	182 (56, 2, 040)		
ART regimen, n (%)			
INSTI-based regimen	28 (74%)	N/A	
NNRTI-based regimen	5 (13%)		
NRTI-based regimen	2 (5.3%)		
Other	3 (7.9%)		
Coinfections, n (%)			N/A
Hepatitis B virus	0 (0%)	0 (0%)	
Hepatitis C virus	0 (0%)	0 (0%)	

^1^ Median (IQR); n (%); ^2^ Wilcoxon rank sum test; ^3^ Pearson’s Chi-squared test; ^4^ Fisher’s exact test.

**Table 2:** Changes in frequencies of CD4 T-cell subsets following SARS-CoV-2 vaccination in PLWH and uninfected controls. Results are presented as median (IQR) of the percentage (frequencies) within total alive CD4 T-cells. Study visits: V1—pre-vaccination baseline, V2—1 month post 1st dose, V3—3 months post 2nd dose, B1—1 month post 3rd dose. Significant differences (*p* < 0.05) are highlighted in bold.

Characteristics	PLWH Longitudinal							Uninfected Longitudinal			PLWH vs. Uninfected Cross-Sectional	
	V1 N = 38 ^1^	V2 N = 38 ^1^	V3 N = 38 ^1^	B1 N = 38 ^1^	V1 vs. V2 ^2^	V1 vs. V3 ^2^	V1 vs. B1 ^2^	V1 N = 24 ^1^	B1 N = 24 ^1^	V1 vs. B1 ^2^	V1 ^3^	B1 ^3^
Memory T-Cell Subsets												
Naive (CD45RA+CD28+CCR7+) CD4 T-cells	19.55 (14.62, 27.73)	26.35 (19.67, 34.05)	29.05 (18.27, 35.15)	28.45 (21.33, 33.70)	**<0.001**	**<0.001**	**<0.001**	11.45 (6.65, 17.12)	21.60 (8.47, 29.63)	**0.001**	**0.002**	**0.017**
Central Memory (CD45RA-CD28+CCR7+) CD4 T-cells	42.35 (35.00, 48.13)	42.60 (37.00, 49.88)	42.90 (38.30, 50.08)	44.10 (37.55, 48.95)	0.14	0.3	0.5	48.05 (40.00, 51.85)	48.20 (39.70, 55.08)	0.2	**0.048**	**0.043**
Transitional Memory (CD45RA-CD28+CCR7-) CD4 T-cells	19.65 (15.50, 22.30)	17.20 (14.83, 20.15)	16.55 (14.33, 19.70)	16.70 (13.55, 21.20)	**0.027**	**0.001**	**<0.001**	20.45 (16.25, 22.65)	16.50 (13.78, 21.20)	**0.075**	0.8	0.9
Effector Memory (CD45RA-CD28-CCR7-) CD4 T-cells	3.23 (1.62, 6.33)	2.00 (1.27, 4.22)	1.68 (1.01, 3.31)	1.65 (0.98, 3.40)	**<0.001**	**<0.001**	**<0.001**	3.98 (2.61, 5.62)	1.37 (0.76, 3.43)	**0.008**	0.5	0.8
Terminally Differentiated (CD45RA+CD28-CCR7-) CD4 T-cells	0.35 (0.12, 0.70)	0.23 (0.11, 0.45)	0.23 (0.07, 0.49)	0.16 (0.08, 0.43)	**0.004**	**<0.001**	**<0.001**	0.28 (0.19, 0.47)	0.12 (0.05, 0.32)	**0.025**	0.7	0.4
T-cell function												
% HLA-DR+CD38+ in CD4 T-cells	2.32 (1.93, 3.67)	2.18 (1.65, 2.86)	2.04 (1.55, 2.69)	2.23 (1.74, 2.96)	**0.010**	**0.016**	**0.013**	2.57 (1.49, 4.92)	2.36 (1.34, 3.83)	0.2	>0.9	0.6
% Ki-67+ in CD4 T-cells	2.70 (2.19, 3.98)	2.30 (2.00, 3.11)	2.32 (1.86, 2.72)	2.39 (1.87, 2.85)	0.074	**<0.001**	**<0.001**	2.36 (1.97, 3.03)	2.12 (1.59, 2.80)	0.3	0.070	0.4
% PD-1+ in CD4 T-cells	18.60 (14.67, 25.33)	19.55 (15.12, 23.98)	22.20 (16.35, 25.65)	20.70 (16.12, 25.97)	0.8	**0.006**	**0.032**	9.32 (6.68, 13.03)	12.15 (11.38, 16.00)	**0.005**	**<0.001**	**<0.001**
% CTLA-4+ in CD4 T-cells	2.96 (2.30, 3.94)	3.16 (2.16, 4.29)	2.73 (2.15, 3.79)	2.70 (2.15, 3.56)	0.2	>0.9	0.7	2.44 (1.90, 3.15)	2.84 (1.87, 3.89)	**0.023**	0.056	0.8
% Senescent (CD28-CD57+) in CD4 T-cells	4.16 (0.98, 9.00)	2.50 (0.70, 6.60)	2.15 (0.71, 4.10)	1.61 (0.85, 4.82)	**<0.001**	**<0.001**	**<0.001**	1.18 (0.37, 3.22)	0.32 (0.13, 3.24)	**0.009**	**0.030**	**0.025**
% CD39+ in CD4 T-cells	4.92 (3.66, 7.37)	4.92 (3.73, 6.81)	5.34 (4.00, 7.69)	5.07 (3.65, 6.98)	0.8	0.6	0.3	2.39 (1.90, 3.60)	2.89 (2.29, 4.36)	**0.037**	**<0.001**	**0.006**
% CD73+ in CD4 T-cells	8.59 (5.22, 12.35)	8.45 (5.77, 12.40)	9.46 (6.10, 13.03)	9.12 (5.98, 13.55)	0.9	**0.048**	**0.005**	11.05 (9.23, 14.12)	12.45 (8.56, 15.95)	0.079	0.062	**0.036**
Chemokine receptors												
% CCR4+ in CD4 T-cells	17.05 (12.20, 22.48)	16.95 (14.90, 23.58)	19.15 (15.85, 22.48)	18.75 (16.72, 22.87)	0.5	**0.006**	0.073	16.35 (6.43, 23.18)	16.25 (10.28, 24.33)	0.2	0.4	0.5
% CCR6+ in CD4 T-cells	38.90 (36.12, 41.20)	38.60 (35.75, 40.25)	39.50 (36.97, 41.25)	39.45 (37.45, 41.25)	0.4	0.7	0.7	29.35 (19.60, 32.05)	31.30 (22.10, 34.67)	0.064	**<0.001**	**<0.001**
% CXCR3+ in CD4 T-cells	19.45 (13.15, 26.20)	17.60 (13.48, 21.32)	15.70 (13.62, 18.17)	16.90 (12.60, 18.77)	0.13	**0.004**	**0.018**	35.40 (25.35, 44.88)	19.40 (12.20, 26.30)	**<0.001**	**<0.001**	0.3
Th subsets												
% Th17 in CD4 T-cells (CD45RA-CCR4+CCR6+ CXCR3-)	4.46 (3.26, 6.66)	4.99 (3.09, 7.38)	6.10 (5.26, 8.09)	6.11 (4.54, 7.16)	0.3	**<0.001**	**0.010**	1.45 (1.07, 2.12)	3.19 (2.19, 4.48)	**<0.001**	**<0.001**	**<0.001**
% Th1-Th17 in CD4 T-cells (CD45RA-CCR4-CCR6+CXCR3+)	5.06 (2.71, 6.87)	3.85 (2.49, 5.55)	3.97 (2.91, 5.03)	4.12 (2.78, 5.41)	**0.030**	**0.007**	0.059	4.28 (2.42, 6.52)	2.68 (2.04, 4.70)	**0.010**	0.6	0.040
% Th2 in CD4 T-cells (CD45RA-CCR4+CCR6-CXCR3-)	6.36 (4.86, 8.41)	7.88 (5.95, 10.06)	8.66 (6.64, 10.31)	9.11 (6.32, 10.40)	0.053	**<0.001**	**0.014**	4.97 (2.86, 9.53)	8.57 (4.80, 10.48)	**0.004**	0.2	0.8
% Th1 in CD4 T-cells (CD45RA-CCR4-CCR6-CXCR3+)	9.97 (5.84, 12.55)	9.00 (5.82, 10.52)	7.26 (5.65, 9.19)	8.14 (5.84, 8.92)	0.2	**0.002**	**0.013**	23.90 (15.40, 27.60)	11.15 (6.63, 16.00)	**<0.001**	**<0.001**	**0.037**
Regulatory T-cells												
% Treg (CD25hi CD127lo FoxP3+) in CD4 T-cells	0.82 (0.52, 1.23)	1.03 (0.76, 1.95)	1.44 (1.15, 2.17)	1.56 (1.11, 1.97)	**0.003**	**<0.001**	**<0.001**	0.64 (0.38, 1.05)	1.21 (0.74, 2.24)	**<0.001**	0.14	0.5
% CD39+ Treg in CD4 T-cells	0.47 (0.28, 0.65)	0.49 (0.30, 0.72)	0.58 (0.43, 0.85)	0.64 (0.34, 0.86)	0.4	**0.002**	**0.003**	0.29 (0.13, 0.49)	0.35 (0.22, 0.88)	**0.004**	**0.032**	0.3
% CD73+ Treg in CD4 T-cells	0.04 (0.02, 0.07)	0.05 (0.03, 0.11)	0.09 (0.05, 0.15)	0.08 (0.05, 0.14)	**0.022**	**<0.001**	**<0.001**	0.05 (0.01, 0.10)	0.13 (0.02, 0.18)	**<0.001**	0.8	0.7
% CD39+CD73+ Treg in CD4 T-cells	0.01 (0.01, 0.03)	0.01 (0.01, 0.04)	0.02 (0.02, 0.05)	0.03 (0.01, 0.04)	0.4	**<0.001**	**0.002**	0.02 (0.00, 0.04)	0.03 (0.01, 0.06)	0.071	0.8	0.6

^1^ Median (IQR); ^2^ Wilcoxon signed rank test; ^3^ Mann–Whitney U test.

**Table 3:** Changes in the frequencies of CD8 T-cell subsets following SARS-CoV-2 vaccination in PLWH and uninfected controls. Results are presented as median (IQR) of the percentage (frequencies) within total alive CD8 T-cells. Study visits: V1—pre-vaccination baseline, V2—1 month post 1st dose, V3—3 months post 2nd dose, B1—1 month post 3rd dose. Significant differences (*p* < 0.05) are highlighted in bold.

Characteristics	PLWH Longitudinal							Uninfected Longitudinal			PLWH vs. Uninfected Cross-Sectional	
	V1 N = 38 ^1^	V2 N = 38 ^1^	V3 N = 38 ^1^	B1 N = 38 ^1^	V1 vs. V2 ^2^	V1 vs. V3 ^2^	V1 vs. B1 ^2^	V1 N = 24 ^1^	B1 N = 24 ^1^	V1 vs. B1 ^2^	V1 ^3^	B1 ^3^
Memory Subsets												
Naive (CD45RA+CD28+CCR7+) CD8 T-cells	8.33 (5.07, 15.63)	12.95 (8.91, 20.60)	15.40 (8.79, 23.02)	15.75 (7.96, 26.08)	**<0.001**	**<0.001**	**<0.001**	8.25 (4.69, 15.15)	20.90 (8.97, 39.67)	**<0.001**	0.8	0.2
Central Memory (CD45RA-CD28+CCR7+) CD8 T-cells	7.70 (4.30, 13.47)	8.49 (5.41, 16.05)	10.90 (6.95, 16.50)	10.90 (6.40, 16.65)	**0.027**	**<0.001**	**0.003**	8.01 (5.77, 10.50)	12.45 (8.38, 14.82)	**<0.001**	0.9	0.6
Transitional Memory (CD45RA-CD28+CCR7-) CD8 T-cells	15.35 (10.60, 17.50)	17.05 (11.28, 21.02)	17.15 (13.12, 20.73)	16.40 (12.22, 21.25)	**0.040**	**0.002**	**0.009**	12.40 (9.21, 14.72)	13.95 (11.88, 20.95)	**0.007**	0.2	0.5
Effector Memory (CD45RA-CD28-CCR7-) CD8 T-cells	24.40 (17.35, 34.98)	20.95 (14.72, 27.80)	15.25 (11.77, 26.12)	17.80 (12.00, 24.70)	**0.009**	**<0.001**	**<0.001**	30.25 (21.68, 35.25)	14.10 (9.51, 24.65)	**<0.001**	0.4	0.5
Terminally Differentiated (CD45RA+CD28-CCR7-) CD8 T-cells	13.90 (8.24, 19.17)	11.90 (7.04, 15.90)	9.93 (6.80, 16.45)	9.82 (7.18, 12.60)	**0.001**	**<0.001**	**0.021**	8.25 (5.56, 11.25)	5.74 (4.45, 8.03)	**0.010**	**0.009**	**<0.001**
T-cell function												
% HLA-DR+CD38+ in CD8 T-cells	3.55 (2.18, 7.49)	3.91 (2.42, 6.18)	3.72 (2.47, 5.25)	3.97 (2.65, 7.11)	0.5	0.7	0.4	3.02 (2.67, 4.22)	3.15 (2.77, 3.88)	0.9	0.4	0.12
% Ki-67+ in CD8 T-cells	1.90 (1.63, 2.55)	2.13 (1.57, 2.66)	1.83 (1.48, 2.24)	1.84 (1.47, 2.46)	0.3	**0.045**	**0.023**	2.20 (1.95, 2.51)	1.95 (1.47, 2.23)	**0.042**	0.2	0.9
% PD-1+ in CD8 T-cells	22.20 (17.20, 27.95)	22.90 (15.57, 28.25)	25.90 (18.68, 31.77)	24.75 (17.45, 30.62)	0.7	**0.011**	**0.045**	16.95 (11.62, 23.92)	17.85 (15.23, 26.67)	**0.037**	0.078	0.12
% CTLA-4+ in CD8 T-cells	1.16 (0.87, 1.52)	1.13 (0.91, 1.41)	1.15 (0.87, 1.40)	1.11 (0.88, 1.57)	0.9	0.4	0.6	1.23 (1.06, 1.59)	1.11 (0.93, 1.39)	0.4	0.4	0.7
% Senescent (CD28-CD57+) in CD8 T-cells	36.90 (25.90, 51.78)	31.20 (23.35, 44.67)	27.60 (17.48, 43.40)	28.50 (18.40, 44.52)	**<0.001**	**<0.001**	**<0.001**	30.00 (15.23, 36.53)	13.45 (9.50, 31.17)	**0.001**	**0.022**	**0.007**
% CD73+ in CD8 T-cells	17.75 (11.53, 31.97)	23.40 (14.95, 32.12)	24.45 (18.42, 39.15)	27.05 (19.02, 38.48)	**0.005**	**<0.001**	**<0.001**	28.80 (22.80, 45.13)	45.10 (35.62, 57.58)	**<0.001**	**0.007**	**<0.001**
% CD39+ in CD8 T-cells	0.93 (0.70, 1.33)	1.00 (0.66, 1.44)	0.95 (0.61, 1.27)	0.88 (0.47, 1.63)	0.5	0.13	0.5	1.41 (0.96, 1.94)	1.97 (1.21, 2.71)	**0.002**	**0.023**	**<0.001**
Chemokine receptors												
% CCR4+ in CD8 T-cells	35.45 (28.53, 46.92)	33.00 (24.93, 43.13)	22.55 (16.70, 28.25)	23.40 (17.42, 35.88)	0.4	**<0.001**	**<0.001**	39.65 (28.15, 52.00)	35.25 (21.50, 46.45)	**0.005**	0.4	0.059
% CCR6+ in CD8 T-cells	28.25 (26.80, 29.35)	28.05 (25.83, 29.73)	27.80 (25.15, 29.58)	28.00 (25.85, 30.05)	>0.9	0.3	>0.9	12.98 (5.14, 23.40)	14.91 (4.63, 25.52)	0.2	**<0.001**	**<0.001**
% CXCR3+ in CD8 T-cells	21.20 (12.85, 36.65)	18.60 (12.85, 26.40)	15.05 (12.05, 19.82)	17.80 (11.47, 22.77)	0.2	**<0.001**	**0.004**	36.55 (30.50, 46.33)	19.55 (13.40, 24.70)	**<0.001**	**<0.001**	0.3

^1^ Median (IQR); ^2^ Wilcoxon signed rank test; ^3^ Mann–Whitney U test.

## Data Availability

The datasets generated during and/or analyzed during the current study are available from the corresponding author on reasonable request.

## Supplementary Materials

The following supporting information can be downloaded at: https://www.mdpi.com/article/10.3390/v15020575/s1, Materials S1: Participant recruitment and inclusion/exclusion criteria; Table S1: List of antibodies used for ex vivo phentonyping of T-cell sunsets; Table S2: List of antibodies used for in vitro cytokine production analysis; Table S3: Spearman rank correlation between participants’ clinical features and SARS-CoV-2-specific CD4 T-cells; Table S4: Spearman rank correlation between participants’ clinical features and SARS-CoV-2-specific CD8 T-cells; Table S5: Changes in the frequencies of CD4 T-cell subsets following SARS-Cov-2 vaccination in PLWH and uninfected controls vaccinated with mRNA SARS-CoV-2 vaccine only; Table S6: Changes in the frequencies of CD8 T-cell subsets following SARS-Cov-2 vaccination in PLWH and uninfected controls vaccinated with mRNA SARS-CoV-2 vaccine only; Figure S1: SARS-CoV-2-specific CD4 and CD8 T-cell response in participants vaccinated with mRNA-type SARS-CoV-2 vaccines only; Figure S2: Frequencies of cytokine producing SARS-CoV-2-specific CD4 T-cells in total CD4 T-cell pool and frequencies of cytokine producing SARS-CoV-2-specific CD8 T-cells in total CD8 T-cell pool at different time points in PLWH and uninfected controls; Figure S3: Examples of gating on CD4 T-cell memory subsets and markers involved in CD4 T-cell function; Figure S4: Examples of gating on CD4 helper T-cell subsets and regulatory T-cells; Figure S5: Examples of gating on CD8 T-cell memory subsets and markers involved in CD8 T-cell function.
