# Supplementary material for: SARS-CoV-2 Vaccine-Induced T-Cell Response after Three Doses in People Living with HIV on Antiretroviral Therapy Compared to Seronegative Controls (CTN 328 COVAXHIV Study)
Source: Viruses. 2023 Feb 19;15(2):575. doi: 10.3390/v15020575 (PMC9959053; doi:10.3390/v15020575)
Supplement: Supplementary file 1 [file viruses-15-00575-s001.zip › CTN328_Supplementary_Materials_1_viruses_submission_9Jan2022.pdf]

SUPPLEMENTARY MATERIALS #1

STUDY DESIGN /METHODS

The CTN 328 study, whose primary objective was to characterize SARS-CoV-2 immunogenicity in PLWH following vaccination, was approved by the McGill University Health Centre Research Ethics Board (REB)(#2022-7857), Ottawa Health Science Network REB (#20210361-01H), University Health Network REB (#21-5504) and the University of British Columbia-Providence Health Care and Simon Fraser University REBs (#H21-01515, #H21-00742). The SSO cohort study, whose primary objective was to characterize natural and vaccine-induced SARS-CoV2 humoral immune response over time, was approved by Ottawa Health Science Network REB (#20200481-01H).

CTN 328 Participants: Sites were encouraged to enrol PLWH previously excluded or underrepresented in early COVID-19 vaccine studies, including those at risk of worse outcomes upon contracting COVID-19 infection: older age (≥55 years, immune non-responders (CD4 T-cell count <350 cells/mm^3, CD4/CD8 <0.75 with undetectable viral load for 1+ year) and multi-morbidity (≥2 comorbidities)[1]. Inclusion criteria included*:* 1) age ≥16 years; 2) having received, or planning to receive, ≥1 dose of COVID-19 vaccine; 3) HIV-seropositive. Exclusion criteria included: 1) Receipt of any blood product or immunoglobulin preparation within 1 month of vaccination; 2) signs or symptoms of active COVID-19 at enrollment. Participants who had received two COVID-19 vaccine doses prior to enrolment were eligible to participate if their 2nd vaccine dose had been administered at most four months post 1st dose.

SSO Participants: A subset of immunocompetent Stop the Spread Ottawa (SSO) participants were used as HIV-negative controls and were included if they had undergone parallel blood collection in relation to vaccination time. A detailed description of this cohort was recently published[2]. Enrollment for the CTN 328 study occurred from April-June 2021 in Vancouver and June 2021-January 2022 for remaining sites. The SSO study enrolled participants from March-August 2021.

Medical history and HIV history: Data were gathered from interviews and clinic chart reviews following written informed consent and included comorbidities, history and date of COVID-19 infection. For PLWH in the CTN328 study, year of HIV diagnosis, CD4 T-cell nadir and medications including ART were also extracted.

REFERENCES

1. Costiniuk CT, Singer J, Langlois MA, Kulic I, Needham J, Burchell A, et al. **CTN 328: immunogenicity outcomes in people living with HIV in Canada following vaccination for COVID-19 (HIV-COV): protocol for an observational cohort study**. *BMJ Open* 2021; 11(12):e054208.

2. Collins E, Galipeau Y, Arnold C, Bosveld C, Heiskanen A, Keeshan A, et al. **Cohort profile: Stop the Spread Ottawa (SSO)—a community-based prospective cohort study on antibody responses, antibody neutralisation efficiency and cellular immunity to SARS-CoV-2 infection and vaccination**. *BMJ Open* 2022; 12(9).
