# Supplementary material for: SARS-CoV-2 Vaccine-Induced T-Cell Response after Three Doses in People Living with HIV on Antiretroviral Therapy Compared to Seronegative Controls (CTN 328 COVAXHIV Study)
Source: Viruses. 2023 Feb 19;15(2):575. doi: 10.3390/v15020575 (PMC9959053; doi:10.3390/v15020575)
Supplement: Supplementary file 1 [file viruses-15-00575-s001.zip › FINAL_Supplementary Figures_viruses_8Feb2023.pdf]

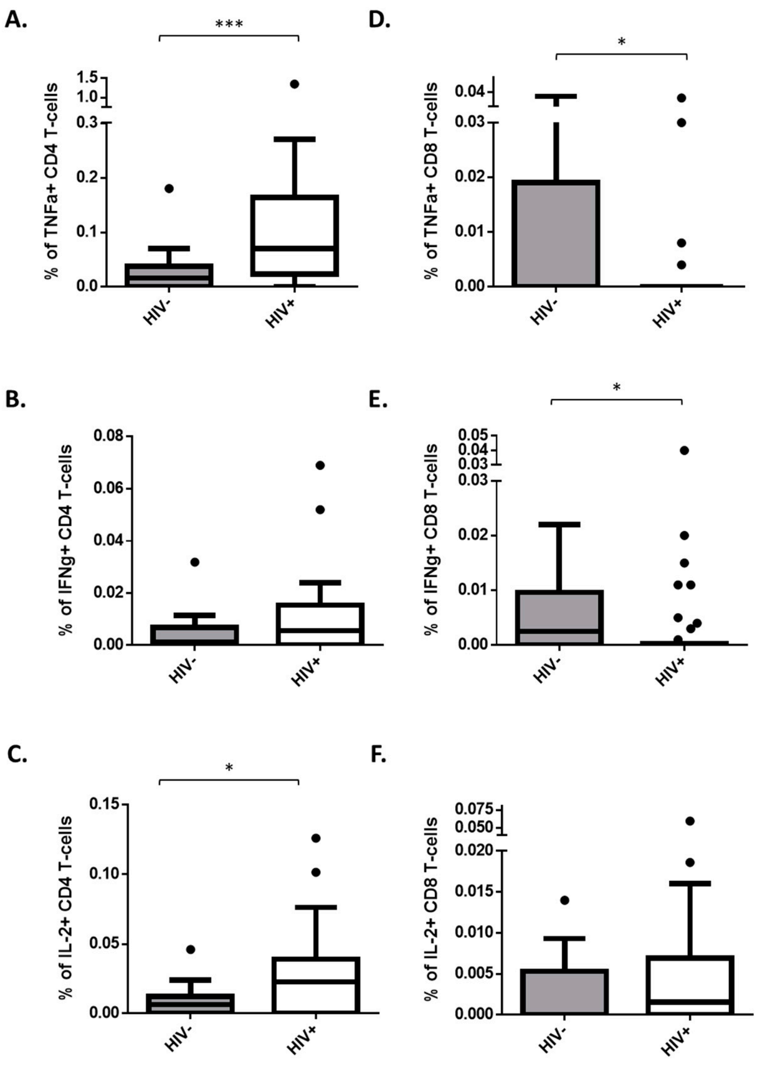

Supplementary Figure S1: (A–C) SARS-CoV-2-specific CD4 and CD8 T-cell response in participants vaccinated with mRNA-type SARS-CoV-2 vaccines only. Frequencies of TNFα+, IFNγ+, and IL-2+ in CD4 T-cells and **(D-F)** CD8 T-cells post 3rd vaccine dose in PLWH *versus* seronegative controls with. Lines in HIV+ vs HIV- plots indicate the median and interquartile range. Comparisons were made using Mann–Whitney U-test (* $p < 0.05$, *** $p < 0.001$).

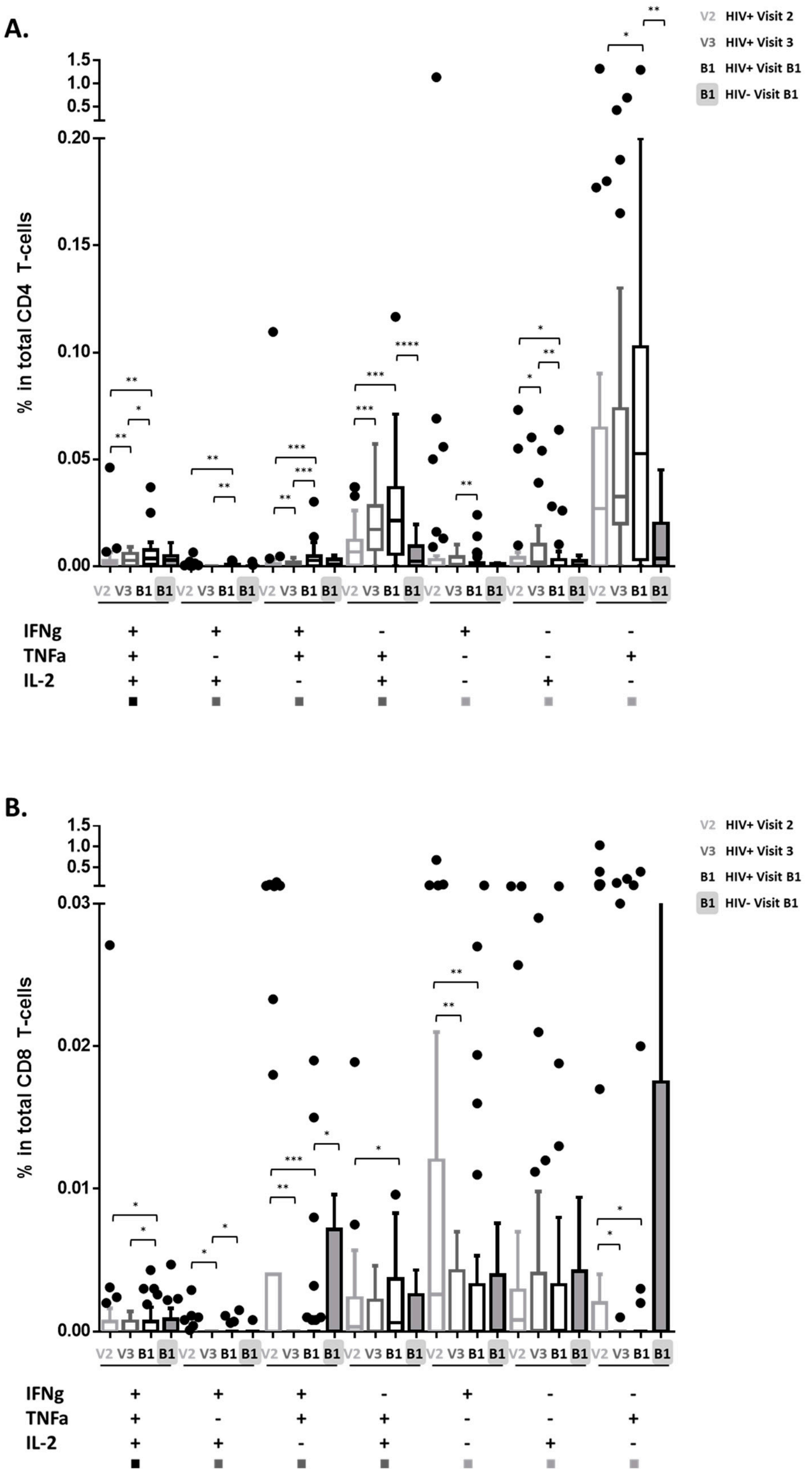

Supplementary Figure S2: (A) Frequencies of cytokine producing SARS-CoV-2-specific CD4 T-cells in total CD4 T-cell pool and **(B)** frequencies of cytokine producing SARS-CoV-2-specific CD8 T-cells in total CD8 T-cell pool at different time points in PLWH and uninfected controls. Lines in boxplots indicate the median and interquartile range. Comparisons were made using Wilcoxon-Pratt signed rank test for paired variables and Mann–Whitney for unpaired variables (* $p < 0.05$, ** $p < 0.01$, *** $p < 0.001$, **** $p < 0.0001$).

A.

B.

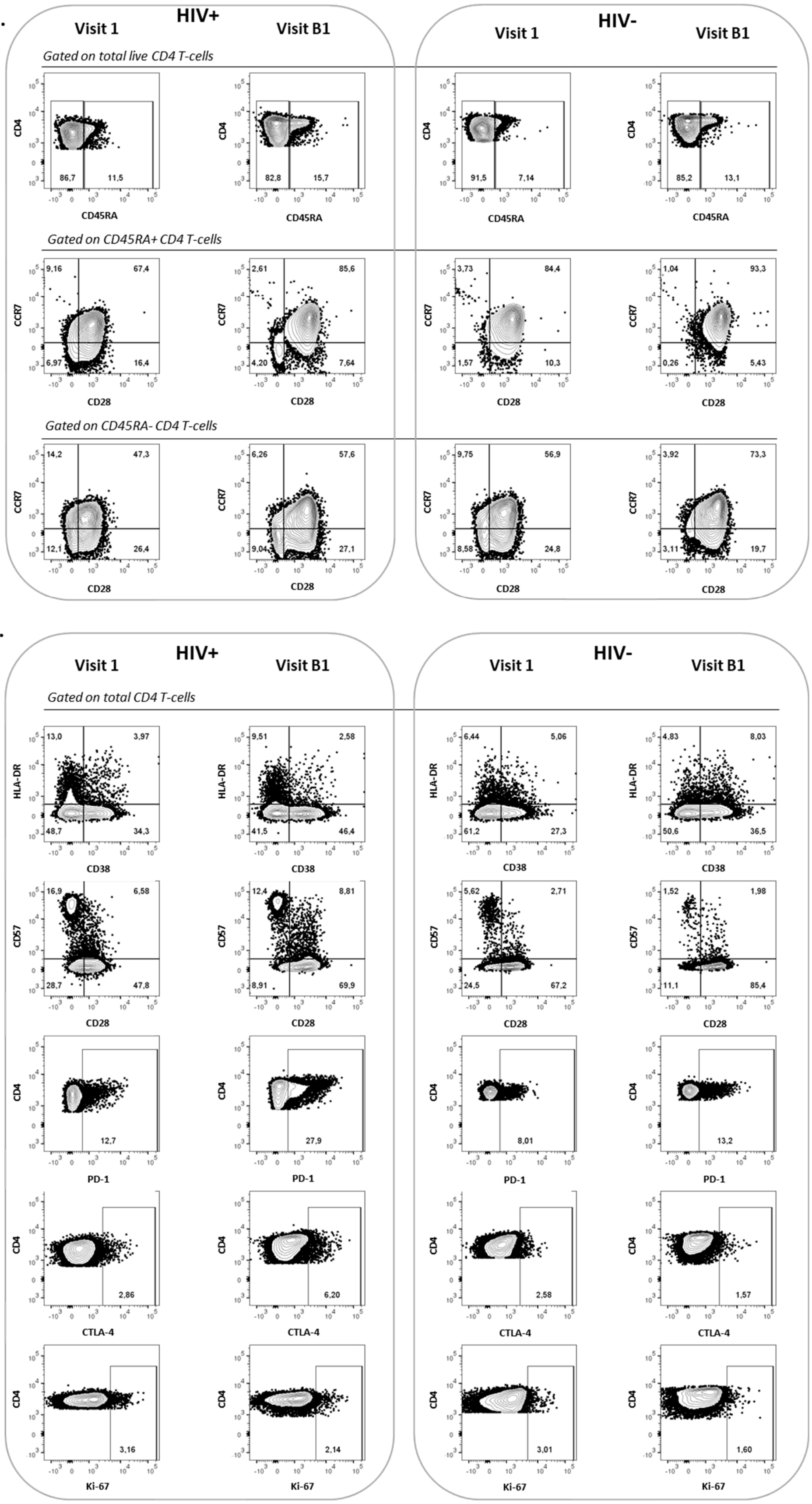

Supplementary Figure S3: (A) Examples of gating on CD4 T-cell memory subsets and **(B)** markers involved in CD4 T-cell function.

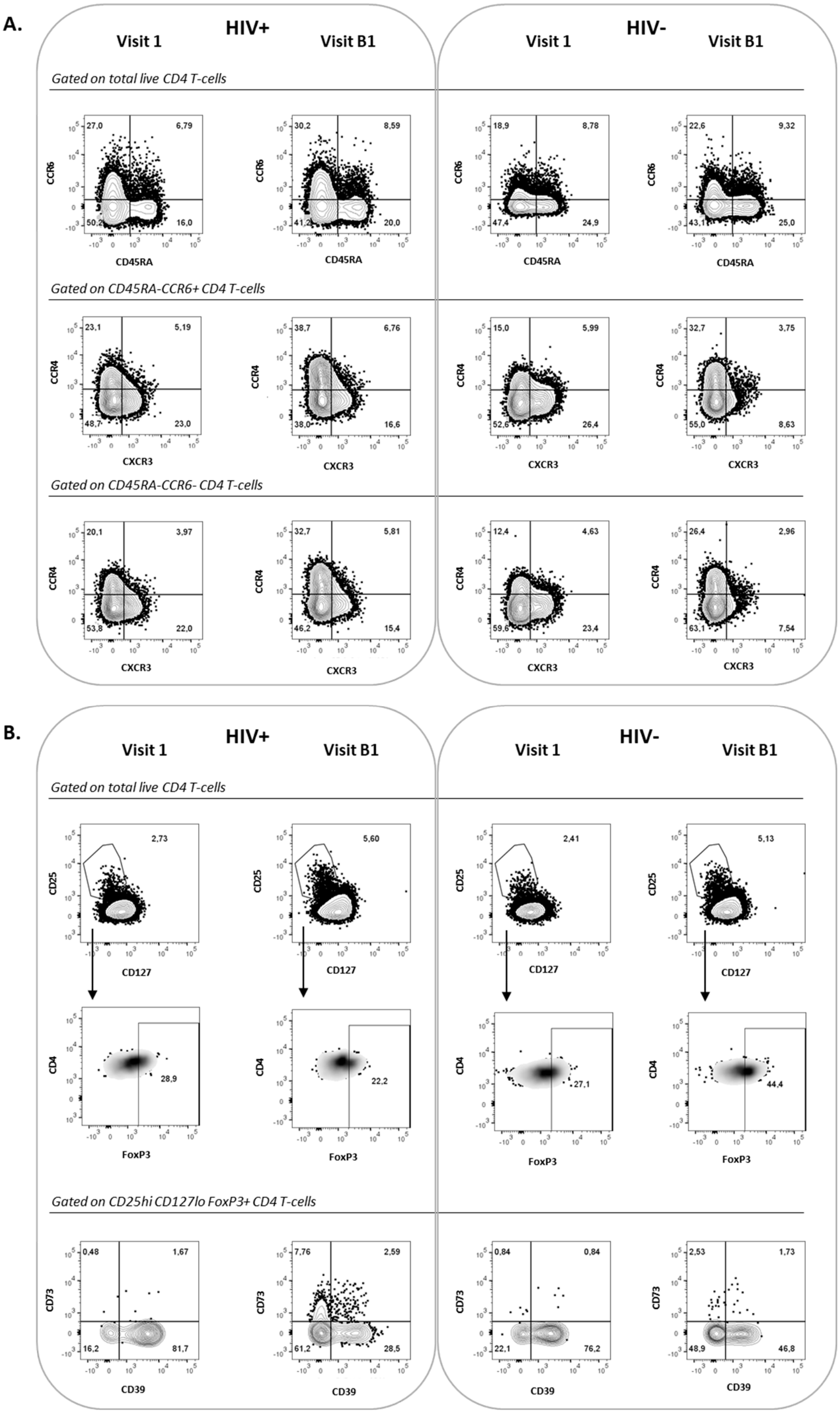

Supplementary Figure S4: (A) Examples of gating on CD4 helper T-cell subsets (Th17: CD45RA-CCR4+CCR6+ CXCR3-; Th1-Th17: CD45RA-CCR4-CCR6+CXCR3+; Th2: CD45RA-CCR4+CCR6- CXCR3-; Th1: CD45RA-CCR4-CCR6- CXCR3+) and **(B)** regulatory T-cells (CD25hi CD127lo FoxP3+).

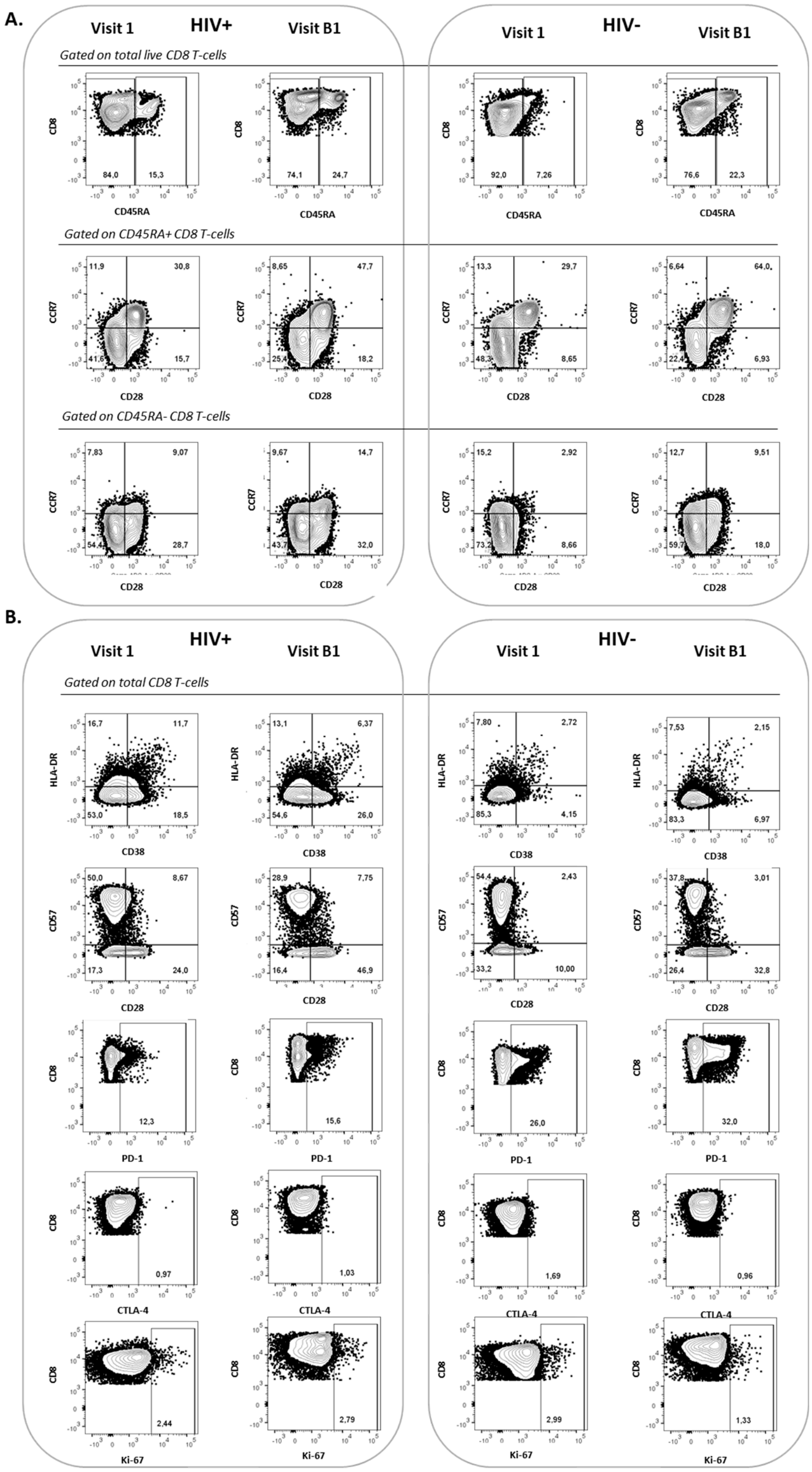

Supplementary Figure S5: (A) Examples of gating on CD8 T-cell memory subsets (Naïve: CD45RA+CD28+CCR7+; Central memory: CD45RA-CD28+CCR7+; Transitional memory: CD45RA-CD28+CCR7-; Effector memory: CD45RA-CD28-CCR7-; Terminally differentiated: CD45RA+CD28-CCR7-) and **(B)** markers involved in CD8 T-cell function.
