# Supplementary material for: SARS-CoV-2 Vaccine-Induced T-Cell Response after Three Doses in People Living with HIV on Antiretroviral Therapy Compared to Seronegative Controls (CTN 328 COVAXHIV Study)
Source: Viruses. 2023 Feb 19;15(2):575. doi: 10.3390/v15020575 (PMC9959053; doi:10.3390/v15020575)
Supplement: Supplementary file 1 [file viruses-15-00575-s001.zip › FINAL_Supplementary Tables_CTN328_viruses_8Feb2023.pdf]

Supplementary Table S1. List of antibodies used for *ex vivo* phentonyping of T-cell subsets.

Ex vivo **T-cell phenotyping**			
**EXTRACELLULAR**			
**mAb**	**Clone**	**Cat. No.**	**Company**
Aqua Vivid 405nm		L34966	Invitrogen
BV786 Mouse Anti-Human CD3	UCHT1	565491	BD Horizon
APC Mouse Anti-Human CD4	RPA-T4	555349	BD Pharmigen
FITC Mouse Anti-Human CD4	RPA-T4	555346	BD Pharmigen
APC-R700 Mouse Anti-Human CD8	RPA-T8	565165	BD Horizon
BV421 Mouse Anti-Human CD194 (CCR4)	1G1	562579	BD Horizon
BB515 Mouse Anti-Human CD196 (CCR6)	11A9	564479	BD Horizon
PE-Cy™5 Mouse Anti-Human CD183 (CXCR3)	1C6/CXCR3	551128	BD Pharmigen
APC-H7 Mouse Anti-Human CD45RA	HI100	561212	BD Pharmigen
PE Mouse Anti-Human CD25	M-A251	555432	BD Pharmigen
PE-Cy™7 Mouse Anti-Human CD127	HIL-7R-M21	560822	BD Pharmigen
BV711 Mouse Anti-Human CD39	TU66	563680	BD Horizon
PerCP-Cy™5.5 Mouse anti-Human CD197	150503	561144	BD Pharmigen
APC Mouse Anti-Human CD28	28.2	559770	BD Pharmigen
BV605 Mouse Anti-Human HLA-DR	G46-6	562845	BD Horizon
PE-Cy™7 Mouse Anti-Human CD38	HIT2	560677	BD Pharmigen
BV421 Mouse Anti-Human CD57	NK-1	563896	BD Horizon
BioLegend PE anti-human CD279 (PD-1)	EH12.2H7	329906	Biolegend
BV605 Mouse Anti-Human CD73	AD2	563199	BD Horizon
**INTRACELLULAR**			
PE-CF594 Mouse Anti-Human FoxP3	236A/E7	563955	BD Horizon
BV711 Mouse Anti-Ki-67	B56	563755	BD Horizon
PE-CF594 Mouse Anti-Human CD152	BNI3	562742	BD Horizon

Supplementary Table S2. List of antibodies used for *in vitro* cytokine production analysis.

In vitro SARS-CoV-2 Spike peptide stimulation			
**EXTRACELLULAR**			
**mAb**	**Clone**	**Cat. No.**	**Company**
Aqua Vivid 405nm		L34966	Invitrogen
BV786 Mouse Anti-Human CD3	UCHT1	565491	BD Horizon
FITC Mouse Anti-Human CD4	RPA-T4	555346	BD Pharmigen
APC-R700 Mouse Anti-Human CD8	RPA-T8	565165	BD Horizon
**INTRACELLULAR**			
PE/Cyanine7 anti-human IFN-γ Antibody	B27	506518	Biolegend
PE-CF594 Mouse Anti-Human TNF	MAb11	562784	BD Horizon
PE Mouse Anti-Human IL-2	MQ1-17H12	12-7029-42	eBioscience

Supplementary Table S3. Spearman rank correlation between participants' clinical features and SARS-CoV-2-specific CD4 T-cells.

CLINICAL FEATURE	% OF TNFα+ IFNγ+ IL-2+ CELLS IN TOTAL CD4 T-CELLS	% OF TNFα+ CELLS IN TOTAL CD4 T-CELLS	% OF IFNγ+ CELLS IN TOTAL CD4 T-CELLS	% OF IL-2+ CELLS IN TOTAL CD4 T-CELLS
**CD4 count**	r=-0.226; p=0.239	r=-0.077; p=0.693	r=-0.084; p=0.666	r=-0.155; p=0.422
**CD4 nadir**	r=-0.195; p=0.312	r=-0.271; p=0.154	r=-0.006; p=0.974	r=0.084; p=0.664
**CD4/CD8 ratio**	r=0.099; p=0.608	r=-0.031; p=0.872	r=0.183; p=0.341	r=-0.021; p=0.915
**Duration of HIV infection (yrs)**	r=0.087; p=0.653	r=0.279; p=0.143	r=0.139; p=0.474	r=0.029; p=0.883

Supplementary Table S4. Spearman rank correlation between participants' clinical features and SARS-CoV-2-specific CD8 T-cells. Significant correlations ($p < 0.05$) are highlighted in bold.

CLINICAL FEATURE	% OF TNFα+ IFNγ+ IL-2+ CELLS IN TOTAL CD8 T-CELLS	% OF TNFα+ CELLS IN TOTAL CD8 T-CELLS	% OF IFNγ+ CELLS IN TOTAL CD8 T-CELLS	% OF IL-2+ CELLS IN TOTAL CD8 T-CELLS
**CD4 count**	r=-0.167; p=0.387	r=-0.097; p=0.618	r=0.052; p=0.789	r=-0.038; p=0.844
**CD4 nadir**	r=0.239; p=0.213	r=-0.137; p=0.478	r=0.070; p=0.718	r=-0.127; p=0.513
**CD4/CD8 ratio**	r=0.079; p=0.685	r=0.065; p=0.737	**r=0.372; p=0.047**	r=0.027; p=0.889
**Duration of HIV infection (yrs)**	r=0.198; p=0.303	r=-0.066; p=0.734	r=0.206; p=0.284	r=0.217; p=0.258

Supplementary Table S5: Changes in the frequencies of CD4 T-cell subsets following SARS-Cov-2 vaccination in PLWH and uninfected controls vaccinated with mRNA SARS-CoV-2 vaccine only. Results are presented as median (IQR) of the percentage (frequencies) within total alive CD4 T-cells. Study visits: V1 - pre-vaccination baseline, V2 - 1 month post 1st dose, V3 - 3 months post 2nd dose, B1 - 1 month post 3rd dose. Significant differences ($p < 0.05$) are highlighted in bold. [1]Median (IQR); [2]Wilcoxon signed rank test; [3]Mann–Whitney U test.

CHARACTERISTICS	PLWH LONGITUDINAL							UNINFECTED LONGITUDINAL			PLWH VS. UNINFECTED CROSS-SECTIONAL	
	**V1** N = 38[1]	**V2** N = 38[1]	**V3** N = 38[1]	**B1** N = 38[1]	**V1 vs. V2**[2]	**V1 vs. V3**[2]	**V1 vs. B1**[2]	**V1** N = 24[1]	**B1** N = 24[1]	**V1 vs. B1**[2]	**V1**[3]	**B1**[3]
**Memory T-cell subsets**												
Naive (CD45RA+CD28+CCR7+) CD4 T-cells	19.55 (14.62, 27.73)	26.35 (19.67, 34.05)	29.05 (18.27, 35.15)	28.45 (21.33, 33.70)	**<0.001**	**<0.001**	**<0.001**	12.35 (7.07, 17.35)	23.10 (9.02, 29.90)	**<0.001**	**0.011**	0.10
Central Memory (CD45RA-CD28+CCR7+) CD4 T-cells	42.35 (35.00, 48.13)	42.60 (37.00, 49.88)	42.90 (38.30, 50.08)	44.10 (37.55, 48.95)	0.14	0.3	0.5	47.55 (40.00, 51.42)	47.90 (39.70, 54.52)	0.3	0.089	0.084
Transitional Memory (CD45RA-CD28+CCR7-) CD4 T-cells	19.65 (15.50, 22.30)	17.20 (14.83, 20.15)	16.55 (14.33, 19.70)	16.70 (13.55, 21.20)	**0.027**	**0.001**	**<0.001**	20.45 (15.12, 22.65)	16.15 (11.90, 19.70)	**0.042**	>0.9	0.7
Effector Memory (CD45RA-CD28-CCR7-) CD4 T-cells	3.23 (1.62, 6.33)	2.00 (1.27, 4.22)	1.68 (1.01, 3.31)	1.65 (0.98, 3.40)	**<0.001**	**<0.001**	**<0.001**	3.90 (2.33, 4.62)	0.95 (0.73, 3.36)	**0.006**	0.8	0.3
Terminally Differentiated (CD45RA+CD28-CCR7-) CD4 T-cells	0.35 (0.12, 0.70)	0.23 (0.11, 0.45)	0.23 (0.07, 0.49)	0.16 (0.08, 0.43)	**0.004**	**<0.001**	**<0.001**	0.28 (0.18, 0.42)	0.12 (0.08, 0.22)	**0.003**	0.5	0.3

**T-cell function**												
% HLA-DR+CD38+ in CD4 T-cells	2.32 (1.93, 3.67)	2.18 (1.65, 2.86)	2.04 (1.55, 2.69)	2.23 (1.74, 2.96)	**0.010**	**0.016**	**0.013**	2.23 (1.45, 4.59)	2.16 (1.33, 3.48)	0.4	0.6	>0.9
% Ki-67+ in CD4 T-cells	2.70 (2.19, 3.98)	2.30 (2.00, 3.11)	2.32 (1.86, 2.72)	2.39 (1.87, 2.85)	0.074	**<0.001**	**<0.001**	2.36 (1.92, 3.03)	2.02 (1.52, 2.74)	**0.040**	0.079	0.2
% PD-1+ in CD4 T-cells	18.60 (14.67, 25.33)	19.55 (15.12, 23.98)	22.20 (16.35, 25.65)	20.70 (16.12, 25.97)	0.8	**0.006**	**0.032**	9.11 (6.68, 13.03)	12.00 (11.18, 14.42)	**0.030**	**<0.001**	**<0.001**
% CTLA-4+ in CD4 T-cells	2.96 (2.30, 3.94)	3.16 (2.16, 4.29)	2.73 (2.15, 3.79)	2.70 (2.15, 3.56)	0.2	>0.9	0.7	2.44 (1.88, 2.98)	2.84 (1.87, 3.58)	0.058	**0.033**	>0.9
% Senescent (CD28-CD57+) in CD4 T-cells	4.16 (0.98, 9.00)	2.50 (0.70, 6.60)	2.15 (0.71, 4.10)	1.61 (0.85, 4.82)	**<0.001**	**<0.001**	**<0.001**	1.03 (0.33, 1.73)	0.24 (0.11, 1.90)	**0.024**	**0.011**	**0.012**
% CD39+ in CD4 T-cells	4.92 (3.66, 7.37)	4.92 (3.73, 6.81)	5.34 (4.00, 7.69)	5.07 (3.65, 6.98)	0.8	0.6	0.3	2.39 (2.12, 3.63)	2.84 (2.29, 3.66)	0.2	**<0.001**	**0.006**
% CD73+ in CD4 T-cells	8.59 (5.22, 12.35)	8.45 (5.77, 12.40)	9.46 (6.10, 13.03)	9.12 (5.98, 13.55)	0.9	**0.048**	**0.005**	11.05 (8.55, 13.68)	12.45 (8.21, 15.95)	0.2	0.11	0.072
Chemokine receptors												

% CCR4+ in CD4 T-cells	17.05 (12.20, 22.48)	16.95 (14.90, 23.58)	19.15 (15.85, 22.48)	18.75 (16.72, 22.87)	0.5	**0.006**	0.073	12.10 (6.36, 23.18)	14.30 (9.89, 22.15)	0.3	0.15	0.13
% CCR6+ in CD4 T-cells	38.90 (36.12, 41.20)	38.60 (35.75, 40.25)	39.50 (36.97, 41.25)	39.45 (37.45, 41.25)	0.4	0.7	0.7	30.45 (19.60, 32.05)	31.65 (21.32, 35.35)	0.2	**<0.001**	**<0.001**
% CXCR3+ in CD4 T-cells	19.45 (13.15, 26.20)	17.60 (13.48, 21.32)	15.70 (13.62, 18.17)	16.90 (12.60, 18.77)	0.13	**0.004**	**0.018**	33.10 (25.35, 41.62)	18.85 (12.20, 24.25)	**<0.001**	**<0.001**	0.5
**Th subsets**												
% Th17 in CD4 T-cells (CD45RA-CCR4+CCR6+ CXCR3-)	4.46 (3.26, 6.66)	4.99 (3.09, 7.38)	6.10 (5.26, 8.09)	6.11 (4.54, 7.16)	0.3	**<0.001**	**0.010**	1.45 (1.01, 2.24)	2.77 (2.17, 4.35)	**0.006**	**<0.001**	**<0.001**
% Th1-Th17 in CD4 T-cells (CD45RA-CCR4-CCR6+CXCR3+)	5.06 (2.71, 6.87)	3.85 (2.49, 5.55)	3.97 (2.91, 5.03)	4.12 (2.78, 5.41)	**0.030**	**0.007**	0.059	3.69 (2.21, 6.86)	2.59 (1.76, 4.70)	**0.007**	0.7	**0.025**
% Th2 in CD4 T-cells (CD45RA-CCR4+CCR6- CXCR3-)	6.36 (4.86, 8.41)	7.88 (5.95, 10.06)	8.66 (6.64, 10.31)	9.11 (6.32, 10.40)	0.053	**<0.001**	**0.014**	4.71 (2.60, 9.66)	8.59 (4.69, 10.48)	**0.030**	0.2	0.7
% Th1 in CD4 T-cells (CD45RA-CCR4-CCR6- CXCR3+)	9.97 (5.84, 12.55)	9.00 (5.82, 10.52)	7.26 (5.65, 9.19)	8.14 (5.84, 8.92)	0.2	**0.002**	**0.013**	22.10 (14.58, 25.30)	11.15 (6.63, 14.47)	<0.001	<0.001	0.10
**Regulatory T-cells**												

% Treg (CD25hi CD127lo FoxP3+) in CD4 T-cells	0.82 (0.52, 1.23)	1.03 (0.76, 1.95)	1.44 (1.15, 2.17)	1.56 (1.11, 1.97)	**0.003**	**<0.001**	**<0.001**	0.62 (0.38, 0.96)	1.16 (0.70, 1.92)	**<0.001**	0.12	0.3
% CD39+ Treg in CD4 T-cells	0.47 (0.28, 0.65)	0.49 (0.30, 0.72)	0.58 (0.43, 0.85)	0.64 (0.34, 0.86)	0.4	**0.002**	**0.003**	0.29 (0.15, 0.49)	0.35 (0.22, 0.74)	**0.010**	**0.044**	0.2
% CD73+ Treg in CD4 T-cells	0.04 (0.02, 0.07)	0.05 (0.03, 0.11)	0.09 (0.05, 0.15)	0.08 (0.05, 0.14)	**0.022**	**<0.001**	**<0.001**	0.05 (0.01, 0.10)	0.13 (0.03, 0.18)	**<0.001**	0.8	0.7
% CD39+CD73+ Treg in CD4 T-cells	0.01 (0.01, 0.03)	0.01 (0.01, 0.04)	0.02 (0.02, 0.05)	0.03 (0.01, 0.04)	0.4	**<0.001**	**0.002**	0.02 (0.00, 0.04)	0.03 (0.01, 0.05)	0.2	0.8	0.6

Supplementary Table S6. Changes in the frequencies of CD8 T-cell subsets following SARS-Cov-2 vaccination in PLWH and uninfected controls vaccinated with mRNA SARS-CoV-2 vaccine only. Results are presented as median (IQR) of the percentage (frequencies) within total alive CD8 T-cells. Study visits: V1 - pre-vaccination baseline, V2 - 1 month post 1st dose, V3 - 3 months post 2nd dose, B1 - 1 month post 3rd dose. Significant differences ($p < 0.05$) are highlighted in bold. [1]Median (IQR); [2]Wilcoxon signed rank test; [3]Mann–Whitney U test.

**CHARACTERISTICS**	**PLWH LONGITUDINAL**							**UNINFECTED LONGITUDINAL**			**PLWH VS. UNINFECTED CROSS-SECTIONAL**	
	**V1** N = 38[1]	**V2** N = 38[1]	**V3** N = 38[1]	**B1** N = 38[1]	**V1** N = 38[1]	**V2** N = 38[1]	**V3** N = 38[1]	**V1** N = 24[1]	**V1** N = 39[1]	**V2** N = 39[1]	**V3** N = 39[1]	**B1**[3] N = 39[1]
**Memory subsets**												
Naive (CD45RA+CD28+CCR7+) CD8 T-cells	8.33 (5.07, 15.63)	12.95 (8.91, 20.60)	15.40 (8.79, 23.02)	15.75 (7.96, 26.08)	**<0.001**	**<0.001**	**<0.001**	9.07 (5.30, 17.40)	25.35 (9.97, 42.77)	**<0.001**	0.8	0.089
Central Memory (CD45RA-CD28+CCR7+) CD8 T-cells	7.70 (4.30, 13.47)	8.49 (5.41, 16.05)	10.90 (6.95, 16.50)	10.90 (6.40, 16.65)	**0.027**	**<0.001**	**0.003**	7.86 (5.77, 10.50)	13.00 (8.38, 14.82)	**<0.001**	0.9	0.5
Transitional Memory (CD45RA-CD28+CCR7-) CD8 T-cells	15.35 (10.60, 17.50)	17.05 (11.28, 21.02)	17.15 (13.12, 20.73)	16.40 (12.22, 21.25)	**0.040**	**0.002**	**0.009**	12.05 (9.21, 13.88)	14.85 (12.30, 20.95)	**0.003**	0.2	0.6
Effector Memory (CD45RA-CD28-CCR7-) CD8 T-cells	24.40 (17.35, 34.98)	20.95 (14.72, 27.80)	15.25 (11.77, 26.12)	17.80 (12.00, 24.70)	**0.009**	**<0.001**	**<0.001**	28.50 (17.47, 33.47)	12.60 (8.63, 21.45)	**<0.001**	0.7	0.13

Terminally Differentiated (CD45RA+CD28-CCR7-) CD8 T-cells	13.90 (8.24, 19.17)	11.90 (7.04, 15.90)	9.93 (6.80, 16.45)	9.82 (7.18, 12.60)	**0.001**	**<0.001**	**0.021**	8.43 (6.05, 11.25)	5.24 (4.45, 7.58)	**0.005**	**0.021**	**<0.001**
**T-cell function**												
% HLA-DR+CD38+ in CD8 T-cells	3.55 (2.18, 7.49)	3.91 (2.42, 6.18)	3.72 (2.47, 5.25)	3.97 (2.65, 7.11)	0.5	0.7	0.4	2.95 (2.51, 3.95)	3.04 (2.72, 3.88)	0.7	0.3	0.089
% Ki-67+ in CD8 T-cells	1.90 (1.63, 2.55)	2.13 (1.57, 2.66)	1.83 (1.48, 2.24)	1.84 (1.47, 2.46)	0.3	**0.045**	**0.023**	2.20 (1.95, 2.58)	1.84 (1.43, 2.15)	**<0.001**	0.14	0.5
% PD-1+ in CD8 T-cells	22.20 (17.20, 27.95)	22.90 (15.57, 28.25)	25.90 (18.68, 31.77)	24.75 (17.45, 30.62)	0.7	0.011	0.045	16.95 (11.62, 22.28)	17.60 (15.23, 26.67)	**0.040**	0.060	0.11
% CTLA-4+ in CD8 T-cells	1.16 (0.87, 1.52)	1.13 (0.91, 1.41)	1.15 (0.87, 1.40)	1.11 (0.88, 1.57)	0.9	0.4	0.6	1.28 (1.12, 1.61)	1.04 (0.88, 1.39)	0.083	0.2	0.5
% Senescent (CD28-CD57+) in CD8 T-cells	36.90 (25.90, 51.78)	31.20 (23.35, 44.67)	27.60 (17.48, 43.40)	28.50 (18.40, 44.52)	**<0.001**	**<0.001**	**<0.001**	23.80 (14.62, 35.27)	11.45 (8.73, 22.00)	**0.002**	**0.009**	**0.001**
% CD73+ in CD8 T-cells	17.75 (11.53, 31.97)	23.40 (14.95, 32.12)	24.45 (18.42, 39.15)	27.05 (19.02, 38.48)	**0.005**	**<0.001**	**<0.001**	29.20 (24.97, 47.55)	51.30 (39.25, 59.45)	**<0.001**	**0.005**	**<0.001**
% CD39+ in CD8 T-cells	0.93 (0.70, 1.33)	1.00 (0.66, 1.44)	0.95 (0.61, 1.27)	0.88 (0.47, 1.63)	0.5	0.13	0.5	1.66 (1.05, 2.10)	2.05 (1.22, 2.71)	**0.009**	**0.008**	**<0.001**
**Chemokine receptors**												

% CCR4+ in CD8 T-cells	35.45 (28.53, 46.92)	33.00 (24.93, 43.13)	22.55 (16.70, 28.25)	23.40 (17.42, 35.88)	0.4	**<0.001**	**<0.001**	34.85 (26.93, 48.55)	35.50 (19.05, 46.45)	**0.035**	0.8	0.086
% CCR6+ in CD8 T-cells	28.25 (26.80, 29.35)	28.05 (25.83, 29.73)	27.80 (25.15, 29.58)	28.00 (25.85, 30.05)	>0.9	0.3	>0.9	18.40 (5.70, 23.52)	22.40 (5.38, 26.52)	0.10	**<0.001**	**<0.001**
% CXCR3+ in CD8 T-cells	21.20 (12.85, 36.65)	18.60 (12.85, 26.40)	15.05 (12.05, 19.82)	17.80 (11.47, 22.77)	0.2	**<0.001**	**0.004**	36.55 (28.70, 46.33)	19.55 (15.52, 24.50)	**<0.001**	**0.001**	0.4
